# Supplementary material for: Extracellular Vesicles from a Helminth Parasite Suppress Macrophage Activation and Constitute an Effective Vaccine for Protective Immunity
Source: Cell Rep. 2017 May 23;19(8):1545–57. doi: 10.1016/j.celrep.2017.05.001 (PMC5457486; doi:10.1016/j.celrep.2017.05.001)
Supplement: Document S1. Supplemental Experimental Procedures and Figures S1–S7 [file mmc1.pdf]

Cell Reports, Volume *19*

## Supplemental Information

# Extracellular Vesicles from a Helminth Parasite Suppress Macrophage Activation and Constitute an Effective Vaccine for Protective Immunity

**Gillian Coakley, Jana L. McCaskill, Jessica G. Borger, Fabio Simbari, Elaine Robertson, Marissa Millar, Yvonne Harcus, Henry J. McSorley, Rick M. Maizels, and Amy H. Buck**

## Figure S1

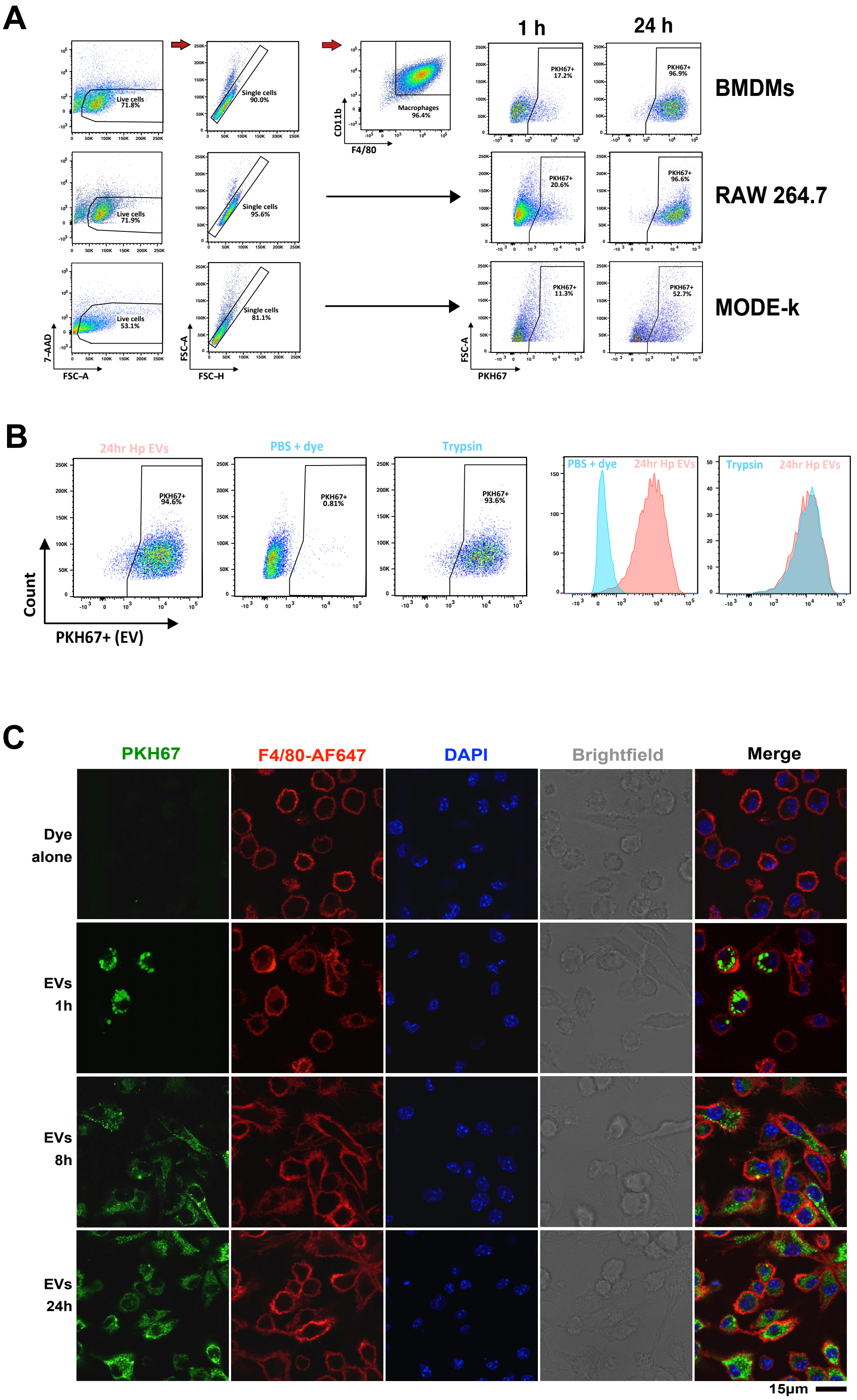


**Supplementary Figure 1 - Related to Figure 1**

(A) Gating strategy for BMDMs (top), RAW 264.7 (middle) cells and MODE-K cells (bottom), in EV uptake assay, isolating the live, single-cell populations (and F480+CD11b+ for macrophage identity), and subsequent uptake of 2.5 µg PHK67-labelled EVs after a 1 h or 24 h. (B) Gating for control assay comparing 24 h incubation of BMDMs with H. polygyrus PKH67-labelled EVs following trypsin treatment (left) or with the PKH67 dye alone (right). (C) Representative single-channel plots of macrophages incubated with PKH67-labelled EVs or dye alone for 1-24 h, DAPI is used to stain nuclei and F4/80-AlexaFlour 647 labelling shows macrophages. Images were taken using a Leica confocal laser-scanning microscope SP5 using a x63 objective; scale bars indicate 15 µm, with representative images shown from 2 independent experiments.

# Figure S2

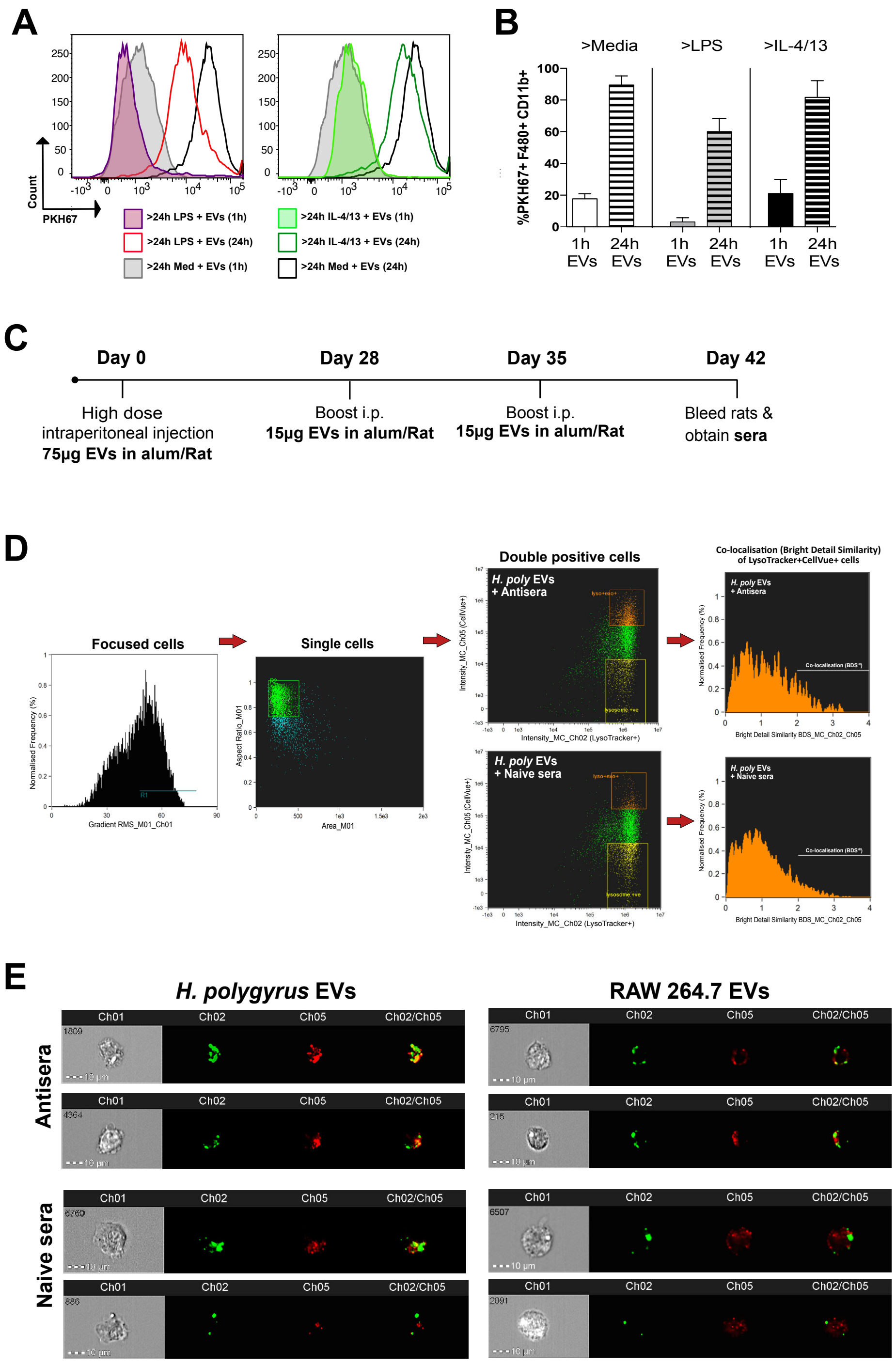


**Supplementary Figure 2 - Related to Figure 2**

(A) Uptake of 2.5 µg EVs was determined after 1 h or 24 h (as indicated) by shift in fluorescence of PKH67 or, (B) the percentage of PHK67+F4/80+CD11b+ cells, in BMDMs pre-treated with 500 ng/ml LPS, 20 ng/ml IL-4/IL-13 or media alone for 24 h. (C) Immunisation schedule for generation of polyclonal anti-EV sera in rats. (D) IDEAS software analysis of acquired BMDMs gated on “cells in focus”, “single cells”, and cellular expression of LysoTracker/CellVue, isolating double positive cells to determine their Bright Detail Similarity (histograms in left panel showing the co-localisation between the fluorescent signals of lysosomes and labelled EVs). (E) Further examples of CellVue-labelled *H. polygyrus* or RAW 264/7-derived EVs incubated with 2.5 x $10^5$ BMDMs (in the presence of naive sera or antisera), as visualised using imagestream analysis. BMDMs incubated with EVs/sera were stained with LysoTracker Green (n=5 biological replicates with 10,000 live cell images taken per replicate).

**Figure S3**

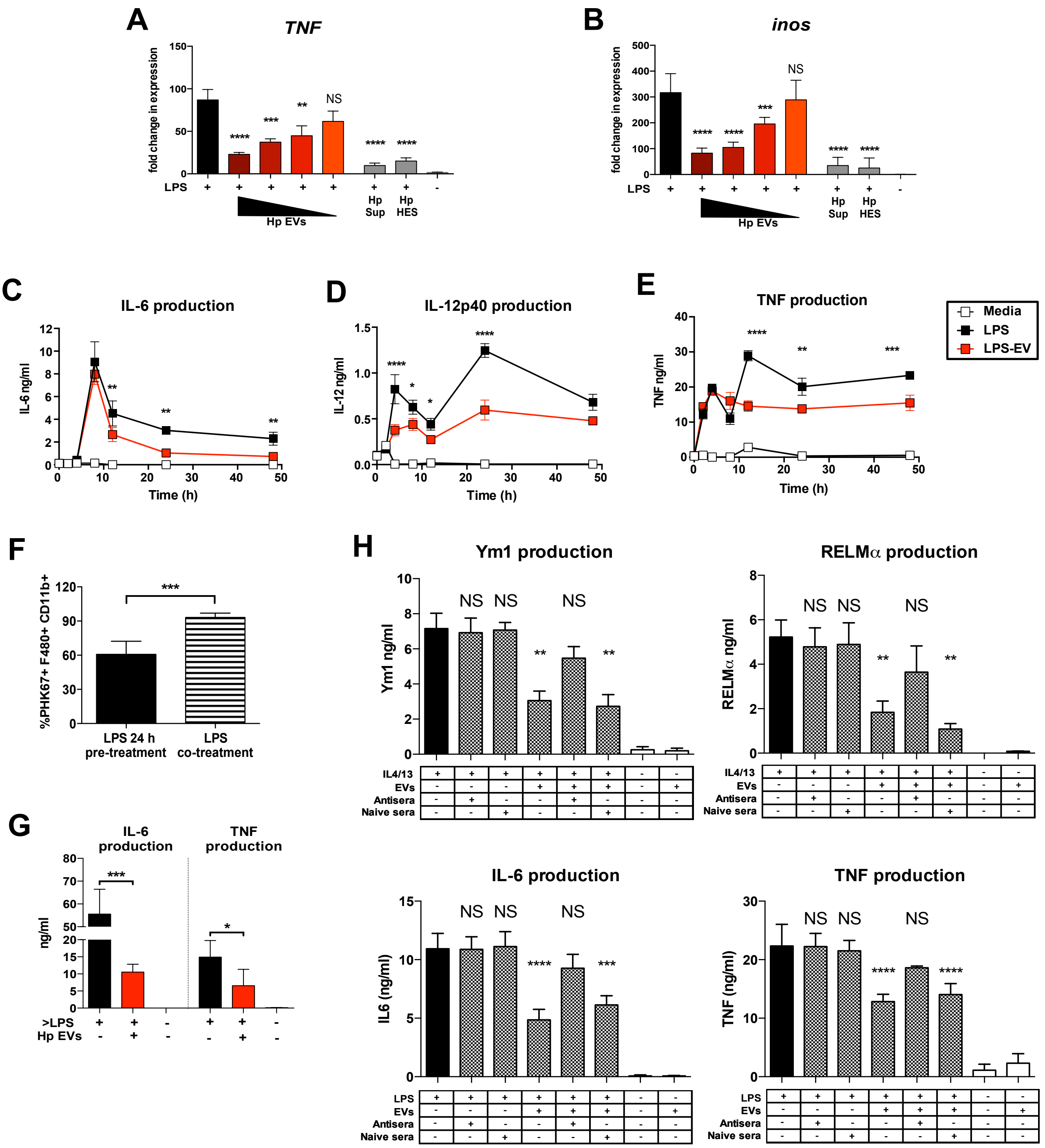


**Supplementary Figure 3 - Related to Figure 3**

(A, B) Relative expression of Tnf and Inos were measured by qRT-PCR and normalized to GAPDH to determine dose dependent effect of EVs (5, 1, 0.5 and 0.25 µg/ml) compared to 5 µg/ml of Sup or HES on LPS stimulation. (C-E) Supernatant levels of IL-6, IL-12p40 and TNF released over 48 h in BMDMs co-treated with 500ng/ml LPS ± 5 µg/ml EVs as measured by ELISA. (F) Uptake of 2.5 µg EVs was determined after 24 h by assessing the percentage of PKH67+F4/80+CD11b+ cells, in BMDMs pre-treated with 500 ng/ml LPS for 24 h, or at time of EV incubation (as indicated). (G) Levels of IL-6 and TNF assessed by ELISA in BMDMs pre-treated for 24 h (indicated by >) or co-treated with 500 ng/ml LPS ± 5 µg/ml EVs or media. (H) Levels of Ym1, RELMα, IL-6 and TNF assessed by ELISA in BMDMs co-treated with IL4/13 or LPS respectively ± 5 µg/ml EVs or media. (A-H) Data are pooled from 2-3 independent experiments (n=6-8) and presented as mean values ± SD; one-way ANOVA for (A-B) + (F-H); two-way ANOVA for (C-E)). * = p<0.05, ** = p<0.01, *** = p<0.001, **** = p<0.0001

## Figure S4

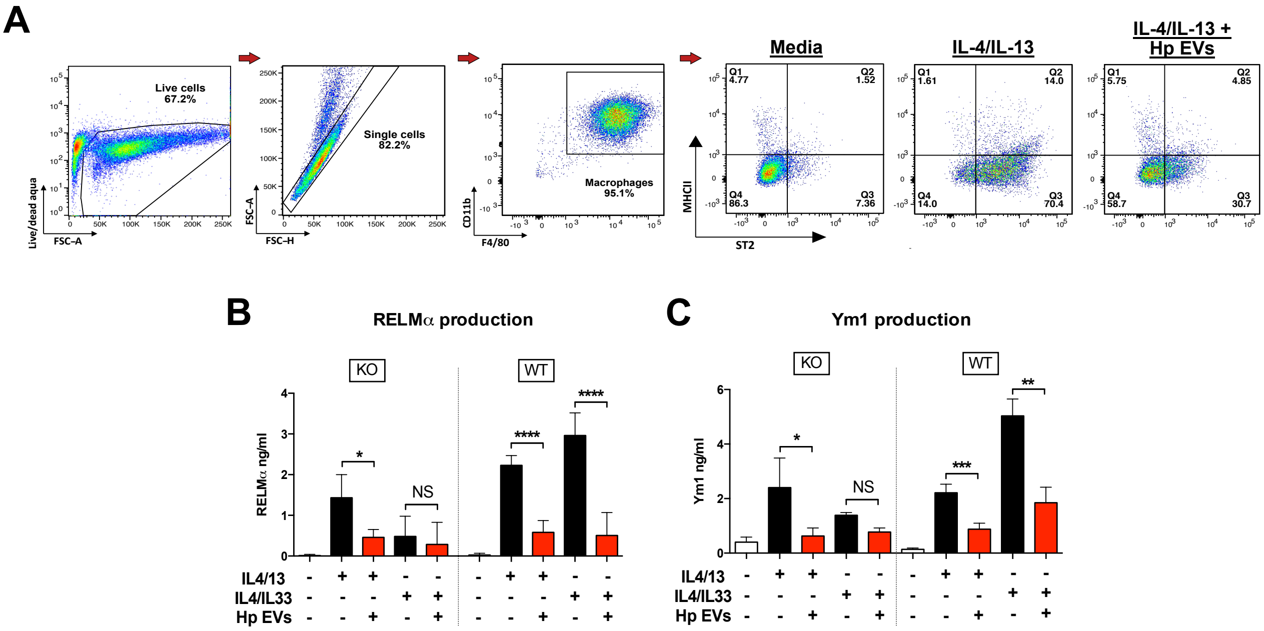


**Supplementary Figure 4 - Related to Figure 4**

(A) Gating strategy for ST2 expression in IL-4/-13 activated macrophages, denoting live cells (live/dead aqua$^-$), exclusion of doublets, macrophages (F4/80$^+$CD11b$^+$), and activation status (ST2 v MHCII). (B) Levels of RELMα and (C) Ym1 found in supernatant from wild type BALB/c and T1/ST2$^{-/-}$ BMDMs treated with 20ng/ml IL-4/IL-33 and/or IL-4/-13 +/- 5 µg/ml EVs for 24 h as measured by ELISA. Data are pooled from 2 independent experiments (n=6) and presented as mean values ± SD; one-way ANOVA. NS indicates a non-significant result. * = p<0.05, ** = p<0.01, *** = p<0.001, **** = p<0.0001.

# Figure S5

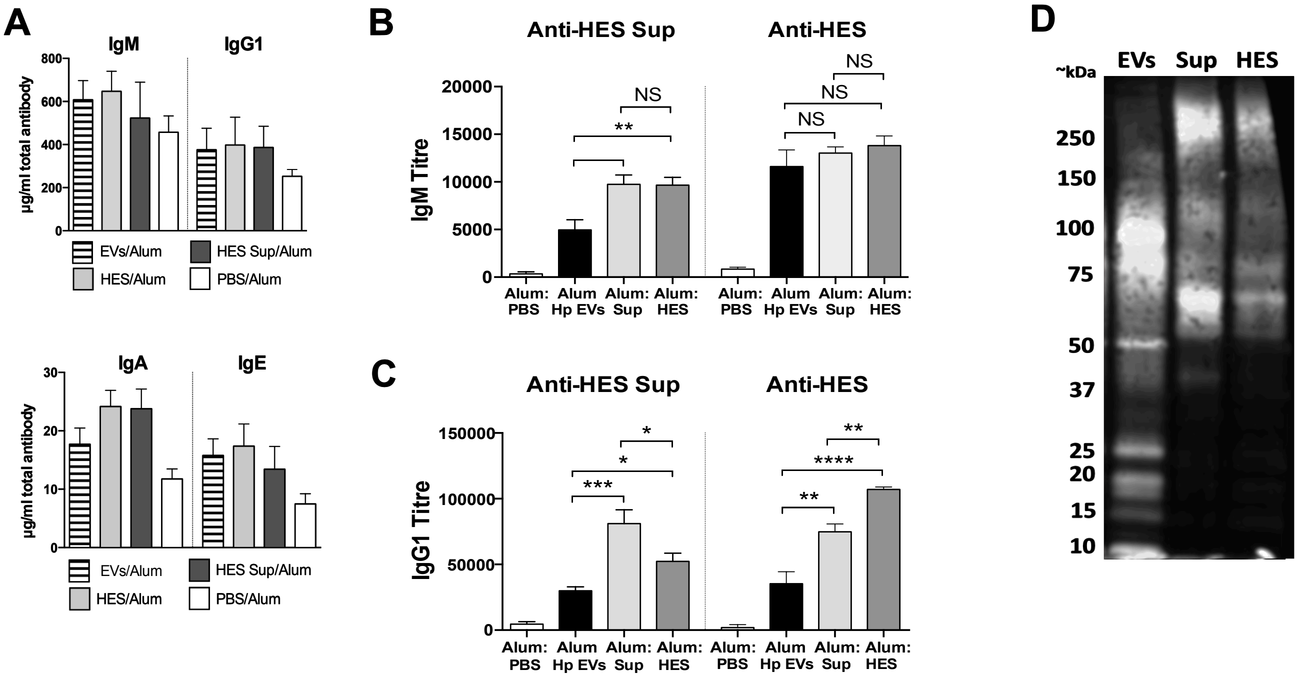


**Supplementary Figure 5 - Related to Figure 5**

Aged matched female C57BL/6 were vaccinated with EVs, Sup, HES or PBS in alum adjuvant (as described in methods). (A) Total serum IgM, IgG1, IgA and IgE levels in each vaccinated group were determined by ELISA against a standard curve of recombinant mouse IgM, IgG1, IgA or IgE. Data are representative from 1 of 2 experiments (n = 5) and presented as mean values ± SD. (B-C) HES-specific and Sup-specific IgM and IgG1 serum titres from EV, Sup, HES or PBS immunized mice (no challenge infection) were measured by ELISA. Data are pooled from 2 experiments and presented as mean values ± SD (n = 10 mice per group). (D) Representative western blot analysis for reactivity of rat polyclonal sera against 2 µg EVs, HES supernatant or total HES, which had first been resolved by SDS-PAGE.

# Figure S6

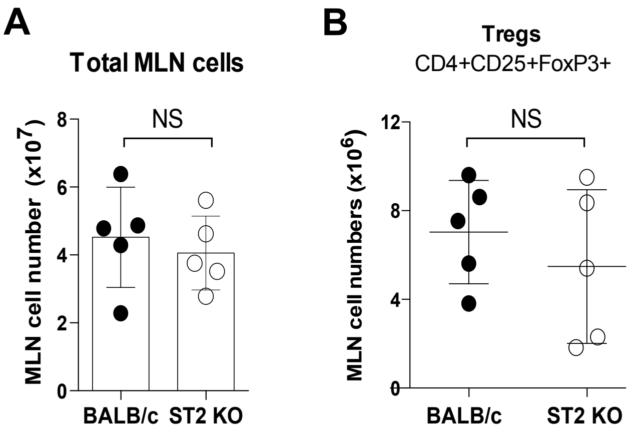


**Supplementary Figure 6 - Related to Figure 6**

MLNs recovered from *H. polygyrus*-infected BALB/c and T1/ST2$^{-/-}$ mice were isolated at day 28 post-infection. (A) Total numbers of MLN cells recovered or (B) absolute numbers of T regulatory cells (CD4+CD25+FoxP3+) were determined by flow cytometry. Data are representative from 1 of 2 experiments and presented as mean values ± SD (n = 5 mice per group; Student's t test). NS indicates a non-significant result.

## Figure S7

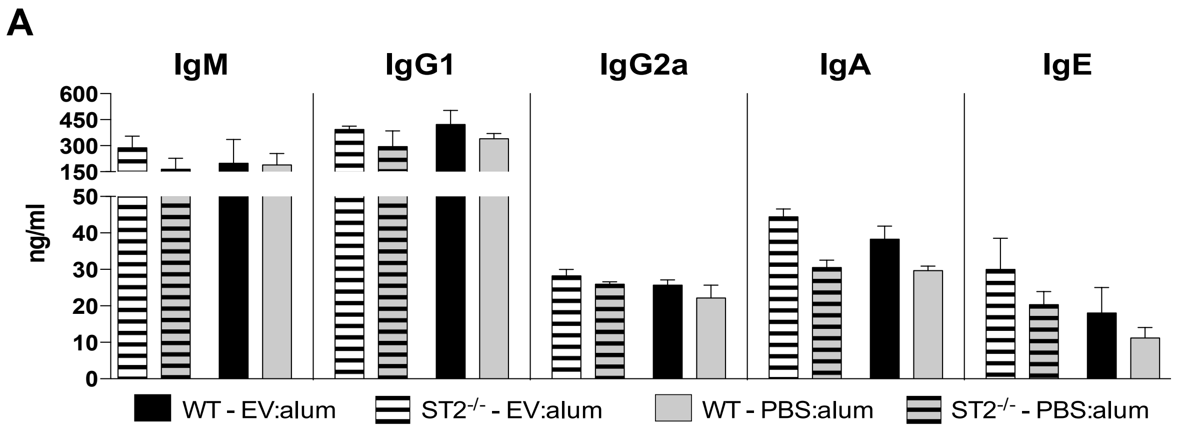


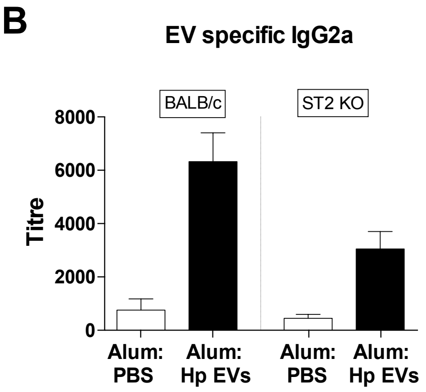


**Supplementary Figure 7 - Related to Figure 7**

Aged matched female BALB/c and T1/ST2$^{-/-}$ mice were vaccinated with EVs or PBS in alum adjuvant (as described in Figure 5A). (A) Total serum IgM, IgG1, IgG2a, IgA and IgE levels in each vaccinated group were determined by ELISA against a standard curve of recombinant mouse IgM, IgG1, IgG2a, IgA or IgE. (B) EV-specific titres of IgG2a detectable from sera of EV or PBS immunized mice measured by ELISA. Data are representative from 1 of 2 experiments and presented as mean values ± SD (n = 4 mice per group).

## Supplemental Experimental Procedures

### Enzyme-linked immunosorbent assay

Supernatants from BMDM culture or *ex vivo* lymphocyte re-stimulation experiments were analysed for levels of IL-13 and IFNγ by ELISA using monoclonal capture and biotinylated detection antibody pairs (BD bioscience). Plates were developed with streptavidin-alkaline phosphatase and p-nitrophenyl phosphate substrate (both Sigma), and read at 405nm.

### Primer list for Quantitative Real-time PCR

| Primer | Target mRNA sequence | 5'- 3' sequence |
|---|---|---|
| ***Arg-1 FWD*** | Arginase | GTC TGT GGG GAA AGC CAA T |
| ***Arg-1 REV*** | | GCT TCC AAC TGC CAG ACT GT |
| ***GAPDH FWD*** | Housekeeping gene | CAT GGC CTT CCG TGT TCC TA |
| ***GAPDH REV*** | | GCG GCA CGT CAG ATC CA |
| ***IL1R1 FWD*** | IL1 receptor-like 1 | AGA CCT GTT ACC TGG GCA AG |
| ***IL1R1 REV*** | | CAC CTG TCT TCT GCT ATT CTG G |
| ***IL6 FWD*** | IL6 cytokine | TGC CTT CAT TTA TCC CTT TGA A |
| ***IL6 REV*** | | TTA CTA CAT TCA GCC AAA AAG CAC |
| ***iNOS FWD*** | Nitric oxide synthase | CAG CTG GGC TGT ACA AAC CTT |
| ***iNOS REV*** | | CAT TGG AAG TGA AGC GTT TCG |
| ***RELMα FWD*** | Resistin-like molecule-α | TAT GAA CAG ATG GGC CTC CT |
| ***RELMα REV*** | | GGC AGT TGC AAG TAT CTC CAC |
| ***TNF FWD*** | Tumour necrosis factor-α | GGA AAT AGC TCC CAG AAA AGC AAG |
| ***TNF REV*** | | TAG CAA ATC GGC TGA CGG TGT G |
| ***Ym1 FWD*** | Murine chitinase | CAT GAG CAA GAC TTG CGT GAC |
| ***Ym1 REV*** | | GGT CCA AAC TTC CAT CCT CCA |

### Western blot analysis

To test the affinity of EV antisera, 5 µg of whole EVs, HES or HES depleted of EVs (HES Sup) were used. Samples were denatured and separated on NuPAGE 4−12% Bis–Tris gels using NuPAGE MES running buffer (Invitrogen) for 35 min at 200 V, before transfer to a nitrocellulose membrane (Biorad) by running in NuPAGE Western buffer (Invitrogen) for 90min at 120 V. Blots were blocked in 5% Milk-PBS with 0.05% Tween 20 for 2 h at RT, before primary polyclonal antisera probe overnight at 4 °C. Using the Licor-based fluorescence detection system, membranes were incubated with a 1:10,000 dilution of Alexa Fluor-680 conjugated to goat anti-Rat IgG (Thermo Fisher Scientific) in 5% milk-PBSt for 45 min, washed in PBS-tween and acquired in PBS using the Odyssey Scanner (Li-Cor Biosciences).
