# Supplementary material for: Extracellular Vesicles from a Helminth Parasite Suppress Macrophage Activation and Constitute an Effective Vaccine for Protective Immunity
Source: Cell Rep. 2017 May 23;19(8):1545–57. doi: 10.1016/j.celrep.2017.05.001 (PMC5457486; doi:10.1016/j.celrep.2017.05.001)
Supplement: Document S2. Article plus Supplemental Information [file mmc2.pdf]

Cell Reports

Article

Extracellular Vesicles from a Helminth Parasite Suppress Macrophage Activation and Constitute an Effective Vaccine for Protective Immunity

Graphical Abstract

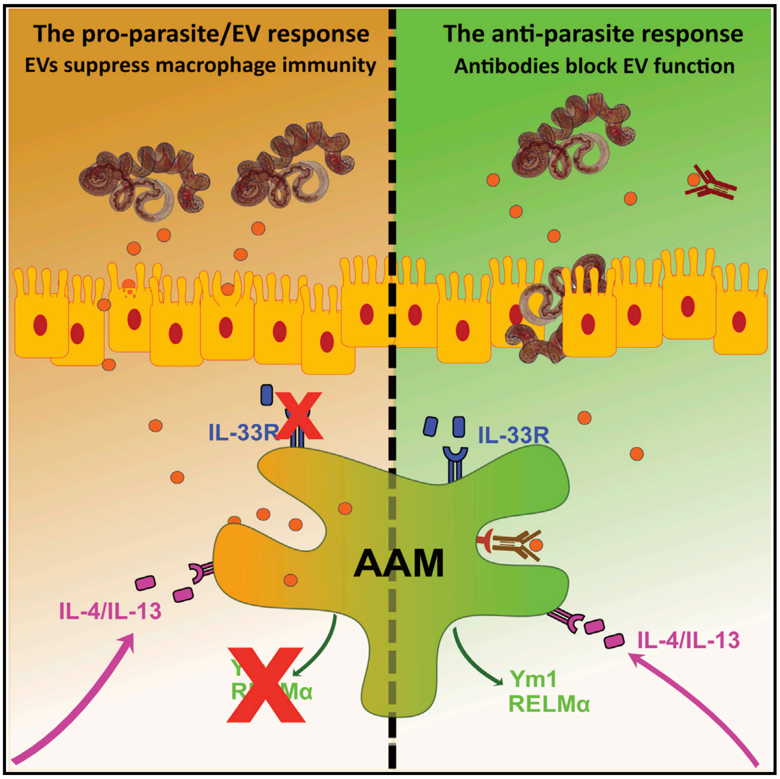

Highlights

- EVs from a nematode parasite suppress type 1 and type 2 activation of macrophages
- Antibodies block EV function and increase their co-localization with the lysosome in macrophages
- EV vaccination generates strong antibody responses and protective immunity against infection
- EVs target both the IL-33 pathway and macrophage activation to counter parasite expulsion

Authors

Gillian Coakley, Jana L. McCaskill, Jessica G. Borger, ..., Henry J. McSorley, Rick M. Maizels, Amy H. Buck

Correspondence

rick.maizels@glasgow.ac.uk (R.M.M.), a.buck@ed.ac.uk (A.H.B.)

In Brief

Coakley et al. find that extracellular vesicles (EVs) from a nematode parasite can suppress host macrophage activation and the alarmin receptor ST2 and that this can be blocked by antibodies. Vaccination with EVs drives strong antibody responses, conferring protection against infection. The authors thus highlight a role for EVs in parasite-host crosstalk.

CrossMark

Coakley et al., 2017, Cell Reports *19*, 1545–1557
May 23, 2017 © 2017 The Author(s).
http://dx.doi.org/10.1016/j.celrep.2017.05.001

CellPress

OPEN ACCESS
CellPress

Extracellular Vesicles from a Helminth Parasite Suppress Macrophage Activation and Constitute an Effective Vaccine for Protective Immunity

Gillian Coakley,[1] Jana L. McCaskill,[1] Jessica G. Borger,[1,4] Fabio Simbari,[1] Elaine Robertson,[1] Marissa Millar,[1] Yvonne Harcus,[1,5] Henry J. McSorley,[2] Rick M. Maizels,[3,6,*] and Amy H. Buck[1,6,7,*]
[1]Institute of Immunology and Infection Research and Centre for Immunity, Infection & Evolution, School of Biological Sciences, University of Edinburgh, Edinburgh EH9 3FL, UK
[2]Centre for Inflammation Research, University of Edinburgh, The Queens Medical Research Institute, 47 Little France Crescent, Edinburgh EH16 4TJ, UK
[3]Wellcome Centre for Molecular Parasitology, Institute for Infection, Immunity and Inflammation, Sir Graeme Davies Building, 120 University Place, Glasgow G12 8TA, UK
[4]Present address: Monash University, Alfred Medical Research and Education Precinct, Level 6 Burnet Tower, 89 Commercial Road, Melbourne, VIC 3004, Australia
[5]Present address: Centre for Cardiovascular Research, University of Edinburgh, The Queen's Medical Research Institute, 47 Little France Crescent, Edinburgh EH16 4TJ, UK
[6]Senior author
[7]Lead Contact
*Correspondence: rick.maizels@glasgow.ac.uk (R.M.M.), a.buck@ed.ac.uk (A.H.B.)
http://dx.doi.org/10.1016/j.celrep.2017.05.001

SUMMARY

Recent studies have demonstrated that many parasites release extracellular vesicles (EVs), yet little is known about the specific interactions of EVs with immune cells or their functions during infection. We show that EVs secreted by the gastrointestinal nematode *Heligmosomoides polygyrus* are internalized by macrophages and modulate their activation. EV internalization causes downregulation of type 1 and type 2 immune-response-associated molecules (IL-6 and TNF, and Ym1 and RELMα) and inhibits expression of the IL-33 receptor subunit ST2. Co-incubation with EV antibodies abrogated suppression of alternative activation and was associated with increased co-localization of the EVs with lysosomes. Furthermore, mice vaccinated with EV-alum generated protective immunity against larval challenge, highlighting an important role in vivo. In contrast, ST2-deficient mice are highly susceptible to infection, and they are unable to clear parasites following EV vaccination. Hence, macrophage activation and the IL-33 pathway are targeted by *H. polygyrus* EVs, while neutralization of EV function facilitates parasite expulsion.

INTRODUCTION

The co-evolution of parasites with their hosts has driven increasingly sophisticated mechanisms of cross-species communication. Recent reports describe the release of extracellular vesicles (EVs) by a broad spectrum of parasites, which may play a central role in this communication (Coakley et al., 2015; Deatherage and Cookson, 2012). EVs can be generated by endocytic pathways or are directly released from the plasma membrane, as documented in the secretions of intracellular *Leishmania spp.* and *Trypanosoma cruzi* parasites (Gonçalves et al., 1991; Silverman et al., 2010). Additionally, EVs are released by extracellular pathogens, providing a mechanism for the import of parasite cargo into host cells, including virulence factors from diverse protozoan parasites, such as *Trypanosoma brucei* and *Trichomonas vaginalis* (Szempruch et al., 2016; Twu et al., 2013).

EVs have also been shown to be a ubiquitous component of metazoan helminth parasite secretions (Chaiyadet et al., 2015; Cwiklinski et al., 2015; Hansen et al., 2015; Marcilla et al., 2012; Nowacki et al., 2015; Tzelos et al., 2016; Zamanian et al., 2015). Helminths are extracellular pathogens that establish long-term chronic infections through the suppression or subversion of host immunity (Coakley et al., 2016; Pearson et al., 2012). A widely used mouse model of chronic helminth infection is the intestinal nematode *Heligmosoides polygyrus*, due to its potent immunoregulatory properties (Behnke et al., 2009; Filbey et al., 2014), which can be largely replicated by excretory-secretory products from this parasite, termed "HES" (McSorley et al., 2014).

We recently discovered that *H. polygyrus* releases exosome-like EVs that are present in HES, suggesting a mechanism for shuttling parasite factors into host cells (Buck et al., 2014). These EVs contain an array of small non-coding RNAs and a specific subset of proteins, and they were shown to modulate murine host gene expression. In particular, the administration of *H. polygyrus* EVs inhibits the activation of type 2 innate lymphoid cells (ILC2) and eosinophils during an allergic airway response

This is an open access article under the CC BY license (http://creativecommons.org/licenses/by/4.0/).

in vivo. Additionally, *H. polygyrus* EVs suppress the receptor for the alarmin cytokine IL-33, in both ILC2s and an intestinal epithelial cell line (Buck et al., 2014).

Binding of IL-33 to the IL-33 receptor (IL-33R, or its subunit, known as T1/ST2, or ST2) is a key interaction that initiates responses in allergy and infection (Molofsky et al., 2015). The release of alarmin cytokines, including IL-33, is closely associated with helminth-mediated tissue damage (Perrigoue et al., 2008; Rostan et al., 2015) and the initiation of type 2 immune responses. A further IL-33-responsive cell is the macrophage, which is strongly polarized to an alternatively activated phenotype following stimulation through IL-33R (Kurowska-Stolarska et al., 2009) and plays a key role in immunity to *H. polygyrus* infection (Anthony et al., 2006; Filbey et al., 2014; Hewitson et al., 2015). Expression of IL-33R is thus associated with host protection from different helminthic diseases. ST2-deficient mice have impaired immune responses with which to challenge *Schistosoma mansoni* (Townsend et al., 2000), *Nippostrongylus brasiliensis*, and *Trichinella spiralis* (Neill et al., 2010; Scalfone et al., 2013), as well as increased susceptibility to a wider range of infectious pathogens (Rostan et al., 2015).

Macrophages and epithelial cells play a central role in driving intestinal immunity to helminths, and, thus, they serve as a prime target for EVs derived from these parasites. In this study, we aimed to understand the mode and function of uptake in these cell types. We demonstrate efficient uptake of EVs by macrophages, which can be functionally blocked by the addition of EV-specific antibodies or inhibitors of actin polymerization. Importantly, the nematode EVs suppress both classical (type 1) and alternative (type 2) activation of macrophages (termed M1 or M2), leading to diminished levels of IL-6, IL-12p40, and TNF, or CD206, CCL17, Ym1, and RELMα, respectively. Independently, nematode EVs suppress expression of the IL-33R in vitro. Interestingly, protective immunity to infection can be induced by vaccination with helminth EVs, but worm expulsion fails in ST2-deficient mice. Hence, the activation of IL-33 signaling is essential for immunity to infection, requiring neutralization of helminth EVs to take place in wild-type mice for parasite expulsion. These data highlight a crucial aspect of cross-phylum communication involving the suppression of macrophage activation and the IL-33 pathway by nematode EVs, and they demonstrate the potential of EVs for vaccination against extracellular helminth parasites.

RESULTS

H. polygyrus EVs Are Efficiently Taken up by Bone Marrow-Derived Macrophages in a Cytochalasin D-Sensitive Manner

To compare the uptake of *H. polygyrus* EVs in epithelial cells and macrophages, they were purified from HES by ultracentrifugation and labeled with PKH67 fluorescent dye, which incorporates into their lipid-rich membrane (Buck et al., 2014). Based on flow cytometry analysis, *H. polygyrus* EV uptake steadily increased over 24 hr in all three cell types examined: F4/80$^+$CD11b$^+$ bone marrow-derived macrophages (BMDMs), the RAW246.7 macrophage cell line, and the MODE-K small intestinal epithelial cell line (Figure 1A; Figure S1A). At both early and late time points, uptake was enhanced in both RAW264.7 and BMDMs (from 14%–23% at 1 hr to 97%–98% at 24 hr) compared to epithelial cells (10% at 1 hr to 55% at 24 hr). Dye was also prepared without EVs to control for any aggregate carryover during purification (<1% of cells were PKH67+ under these conditions; Figure S1B). Additionally, cells were treated with trypsin 5 min prior to acquisition to remove any vesicles bound non-specifically to the cell surface (no differences observed; Figure S1B). *H. polygyrus* EV uptake over time was also verified in BMDMs by confocal microscopy (Figure 1B; Figure S1C). To test whether uptake was occurring by an active process, we co-treated BMDMs with EVs and cytochalasin D, a potent inhibitor of actin polymerization that blocks endocytosis and phagocytosis and prevented EV uptake in other studies (Escrevente et al., 2011; Kusuma et al., 2016). Cytochalasin D treatment blocked EV uptake at 1 and 24 hr post-incubation, with 1% and 8% of cells being PKH67+, respectively (Figures 1C and 1D).

EV Uptake Is Modulated by Macrophage Polarization and the Presence of Specific Antibodies

We next investigated whether polarization of macrophages to either an M1 or M2 phenotype affects parasitic EV uptake, as both cell types are involved in the response to *H. polygyrus* and translocating bacteria during parasite invasion (Weng et al., 2007). BMDMs were pre-treated with lipopolysaccharide (LPS), IL-4/IL-13, or media alone for 24 hr prior to the addition of *H. polygyrus* EVs for 1 hr, and vesicle uptake was assessed by flow cytometry (Figure 2A). LPS pre-treatment significantly repressed the ability of BMDMs to take up parasitic EVs after 1 hr (4%), compared to either naive BMDMs (17%) or those polarized by IL-4/IL-13 (21%). These data are consistent with reports showing superior nanoparticle uptake in M2-polarized macrophages (Hoppstädter et al., 2015). Additionally, LPS depression of EV uptake suggests that early internalization may occur by phagocytosis, which is inhibited in LPS-stimulated macrophages (Feng et al., 2011). Although LPS pre-stimulation limited the initial uptake of EVs in BMDMs, 60% of LPS-stimulated cells were PKH67+ after 24 hr, compared to cells pre-treated with IL-4/IL-13 (82%) or media alone (90%) (Figures S2A and S2B).

To determine if uptake could be blocked by anti-EV antibodies, BMDMs were stimulated with LPS, IL-4/IL-13, or media for 1 hr in the presence of polyclonal antisera (from rats immunized with EVs in alum adjuvant; Figure S2C). Interestingly, antisera treatment significantly enhanced uptake of parasite-derived EVs in all cases (Figure 2B). We also noted a more dispersed pattern of parasite-derived vesicles within antiserum-treated cells after 1 hr (Figure 2C), suggesting that their biological fate may be altered by these conditions.

To analyze the intracellular destination of EVs taken up in the presence or absence of specific antibodies, we adopted imaging flow cytometry to compare localization of labeled EVs and lysosomes (LysoTracker) in a large number of cells (Heusermann et al., 2016). As shown in Figures 2D, S2D, and S2E, the addition of anti-EV sera increased co-localization of *H. polygyrus* EVs with LysoTracker, compared to treatment with control naive serum or when EVs from RAW264.7 cells were tested. By collecting data from 50,000 cells, antisera treatment was shown to result in a

OPEN ACCESS
CellPress

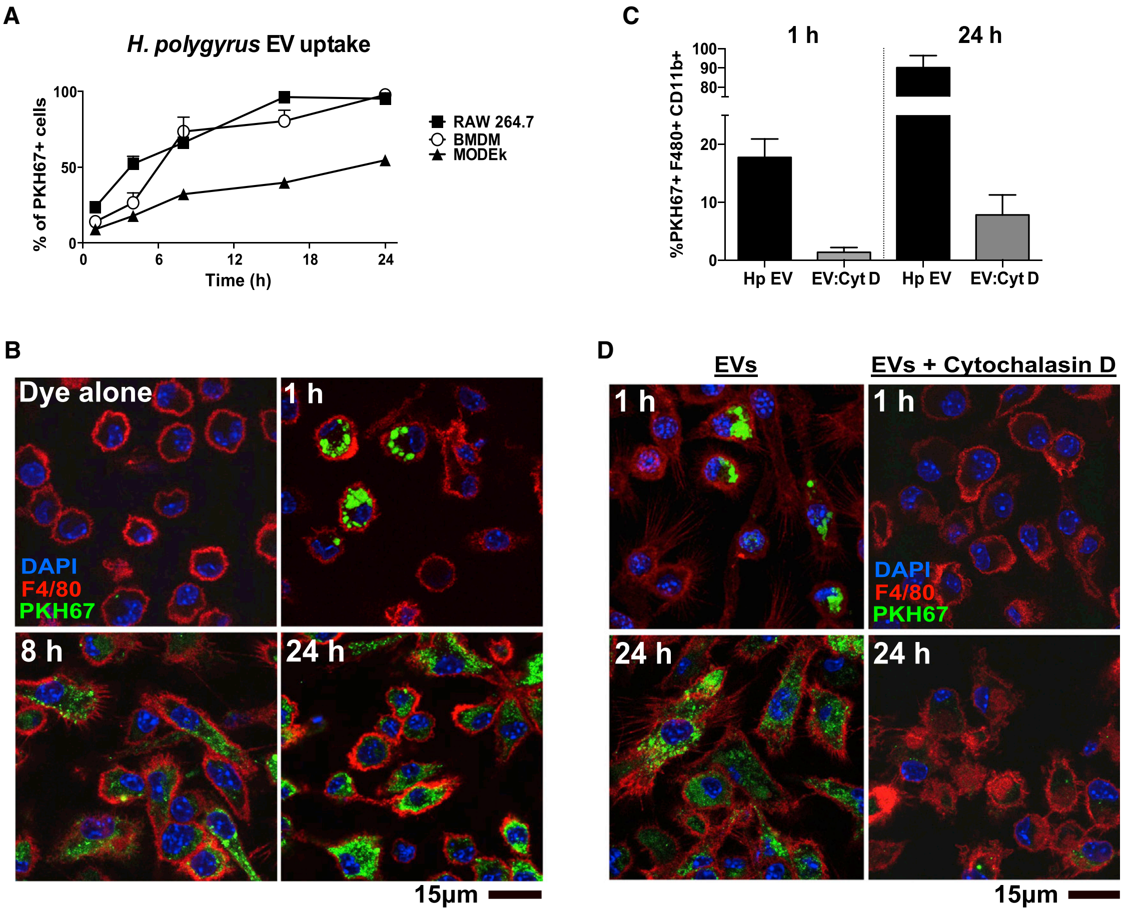

Figure 1. *H. polygyrus* EVs Are Efficiently Taken up by Bone Marrow-Derived Macrophages in a Cytochalasin D-Sensitive Manner

(A) Percentage uptake of 2.5 μg PKH67-labeled EVs incubated with 2.5 × 10^5 BMDMs, RAW264.7, or MODE-K cells from 1 to 24 hr, as assessed by flow cytometry using the gating strategy in Figure S1A. Data are presented as mean values ± SD (n = 3).

(B) Confocal microscopy image of PKH67-labeled EVs incubated with BMDMs from 1 to 24 hr. DAPI is used to stain nuclei and F4/80-Alexa Fluor-647 to label macrophages.

(C) Percentage uptake of 2.5 μg PKH67-labeled EVs (Hp EVs) after 1 or 24 hr in the absence or presence of 4 hr pre-treatment of BMDMs with 2 μg/mL cytochalasin D, as assessed by flow cytometry. Data are presented as mean values ± SD (n = 5).

(D) Confocal microscopy image of the cells treated as in (C) using the fluorescent markers described for (B). For (B) and (D), scale bars represent 15 μm, with representative images shown.

significant increase in the co-localization of *H. polygyrus* EVs with lysosomes (Figure 2E).

H. polygyrus EVs Suppress the Onset of Alternative and Classical Activation in Macrophages

Given the importance of M2 macrophages in mediating parasite expulsion (Kreider et al., 2007), we investigated whether EVs interfere with alternative activation, as we had previously shown that intranasal *H. polygyrus* EV treatment in an allergy model limited activation of ILC2s in the lung (Buck et al., 2014). We therefore tested the effects of EVs on the alternative activation of BMDMs (termed AAMϕ) during a 24-hr co-culture with IL-4 and IL-13. We included for comparison both total HES or HES supernatant (HES depleted of EVs by ultracentrifugation). As a further control, cells were treated with EVs derived from mouse epithelial cells. We observed a marked ablation in the transcriptional hallmarks of AAMϕ (Rückerl and Allen, 2014), namely, resistin-like molecule alpha (RELMα), Ym1, and Arginase 1 (Figure 3A). This suppression was also reflected in levels of RELMα, Ym1, and the M2-associated chemokine CCL17 released into culture supernatant, which were significantly reduced following EV co-treatment (Figure 3B). The comparative suppression of both the mRNA transcripts and protein production by the supernatant and HES suggest that there are other factors in HES (excluding the EV fraction) that also modulate the alternative activation of macrophages. The ability of helminth-derived EVs to suppress AAMϕ suggests that the release of *H. polygyrus*-derived EVs during infection may restrain anti-parasite host responses by macrophages.

To ascertain whether helminth EVs can also suppress the function of cells after the onset of alternative activation, they were added to BMDMs 24 hr following IL-4/IL-13 pre-treatment. Subsequent production of RELMα and Ym1 were markedly

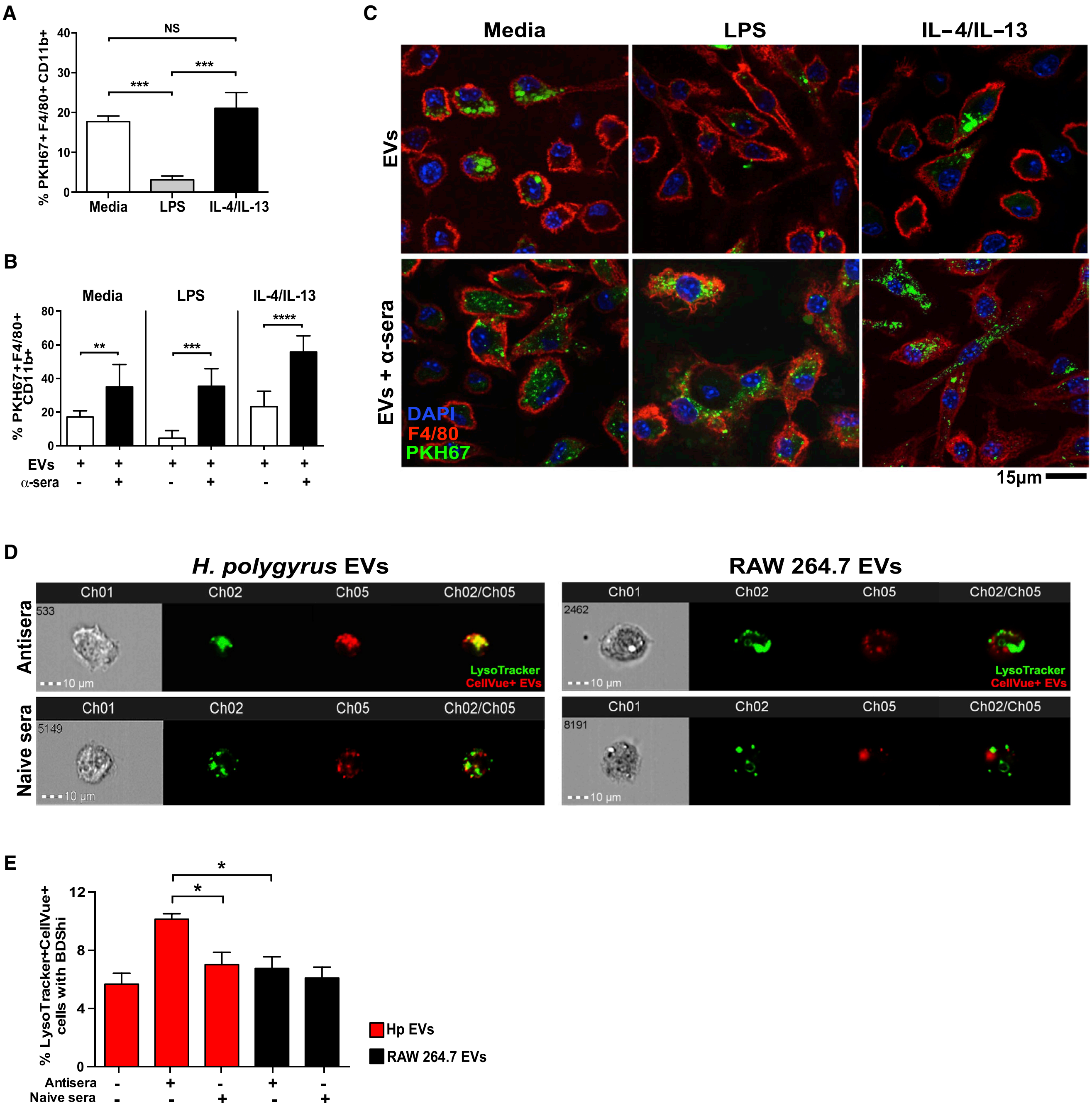

Figure 2. EV Uptake Is Modulated by Macrophage Polarization and the Presence of Specific Antibodies

(A) Percentage uptake of 2.5 μg PHK67-EVs into 2.5 × 10^5 BMDMs that were pre-treated for 24 hr with media alone, 500 ng/mL LPS, or 20 ng/mL IL-4/IL-13, as assessed by flow cytometry using the gating strategy in Figure S1A.

(B) Quantification of uptake as in (A) but comparing incubation of PHK67-EVs ± polyclonal EV antisera (1:2,000). Data were analyzed 1 hr after incubation and are presented as mean values ± SD (n = 5–7; one-way ANOVA, *p < 0.05, **p < 0.01, ***p < 0.001, and ****p < 0.0001; NS, not significant).

(C) Confocal microscopy of PHK67-labeled EVs incubated with BMDMs as described in (B).

(D) Imagestream analysis measuring uptake of 2.5 μg CellVue-labeled *H. polygyrus* or RAW264/7-derived EVs ± polyclonal parasite-EV antisera or naive rat sera (both 1:2,000). BMDMs incubated with EVs/sera were also stained with LysoTracker Green (n = 5; 10,000 live-cell images taken per replicate).

(E) Quantitation of cells showing high co-localization (represented as a measure of bright detail similarity) of LysoTracker with CellVue-labeled EVs in double-positive cells, as determined by IDEAS software. Unless indicated, scale bars represent 15 μm, with representative images shown.

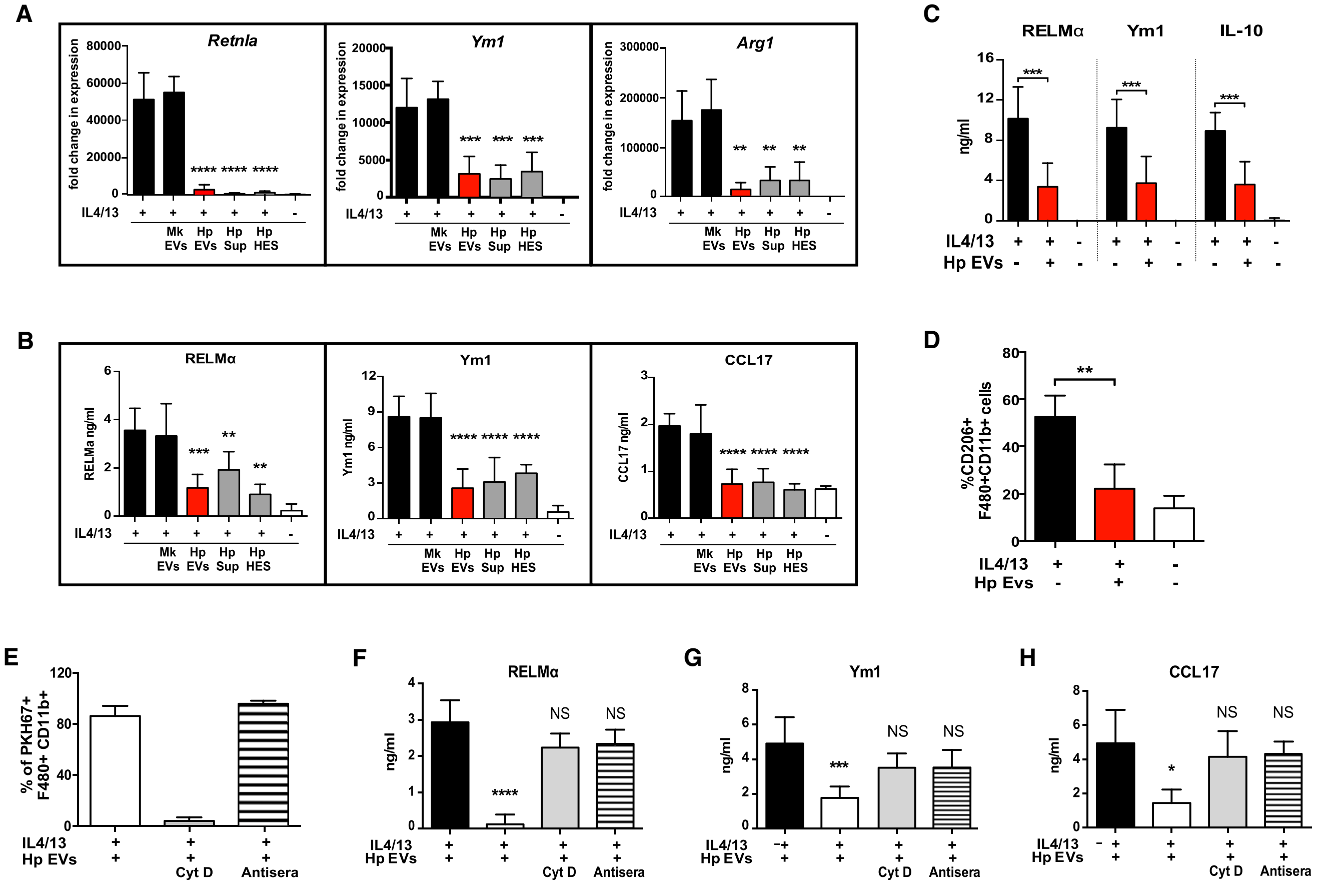

Figure 3. *H. polygyrus* EVs Suppress the Onset of Alternative Activation in Macrophages

(A and B) Relative expression of (A) markers of alternative activation Retnla, Ym1, and Arg1 based on qRT-PCR analysis of extracted RNA or levels of (B) RELMα, Ym1, and CCL17 proteins measured by ELISA from BMDMs treated with 20 ng/mL IL-4/IL-13 ± 5 μg/mL EVs, EV-depleted HES (sup), or total HES (HES) or media alone for 24 hr. As a comparison, cells were incubated with 5 μg/mL EVs derived from MODE-K cells (MK).

(C and D) The levels of (C) RELMα, Ym1, and IL-10 by ELISA and expression of (D) CD206 by flow cytometry in BMDMs that were pre-treated with IL-4/IL-13 for 24 hr prior to the addition of 5 μg/mL EVs.

(E–H) Percentage uptake of (E) 2.5 μg PHK67-labeled EVs was determined by flow cytometry, and levels of (F) RELMα, (G) Ym1, and (H) CCL17 in the supernatant were assayed from BMDMs co-treated with IL-4/IL-13 and EVs ± 2 μg/mL cytochalasin D or EV antisera (1:2,000) for 24 hr. All data are pooled from two to three independent experiments and presented as mean values ± SD (n = 5–9; one-way ANOVA, *p < 0.05, **p < 0.01, ***p < 0.001, and ****p < 0.0001; NS, not significant).

reduced, as was the release of IL-10 and surface expression of CD206 (the mannose receptor), further corollaries of alternative activation (Figures 3C and 3D). Thus, parasite EVs retain their suppressive properties even in cells previously primed with alternatively activating cytokines.

To determine whether the suppressive effects extended to more broadly suppress activation of macrophages, we also tested EVs in classical (LPS-driven) activation, as it is thought that helminth excretory-secretory products (ES) counteract the inflammatory consequences of their damage to the epithelial cell barrier (Gause et al., 2013). We found that *H. polygyrus*-derived EVs also suppressed, in a dose-dependent manner, the hallmarks of LPS-activated BMDMs, including mRNA for IL-6, inducible nitric oxide synthase (iNOS), and tumor necrosis factor (TNF) at 24 hr co-culture (Figures S3A and S3B) and IL-6, IL-12p40, and TNF protein secretion at 48 hr (Figures S3C–S3E). EVs were also able to significantly suppress TNF and IL-6 release in BMDMs that had undergone LPS pre-treatment (Figures S3F and S3G).

Antibody-Mediated EV Uptake Blocks the Inhibition of Alternative Activation

We then sought to determine if there were functional repercussions from antibody interference with EV uptake. Although there was increased uptake of EVs in AAMϕ in the presence of polyclonal sera after 1 hr, the differences were not detected by 24 hr, suggesting antibodies may opsonize EVs for more rapid uptake. This is in contrast to the effects of cytochalasin D-treated cells, where uptake was blocked at both 1 and 24 hr (Figures 1C and 3E). Importantly, both antisera and cytochalasin D abrogated the functions of EVs, as reflected by the reduced ability of the AAMϕ to produce RELMα, Ym1, and CCL17 following

OPEN
ACCESS
CellPress

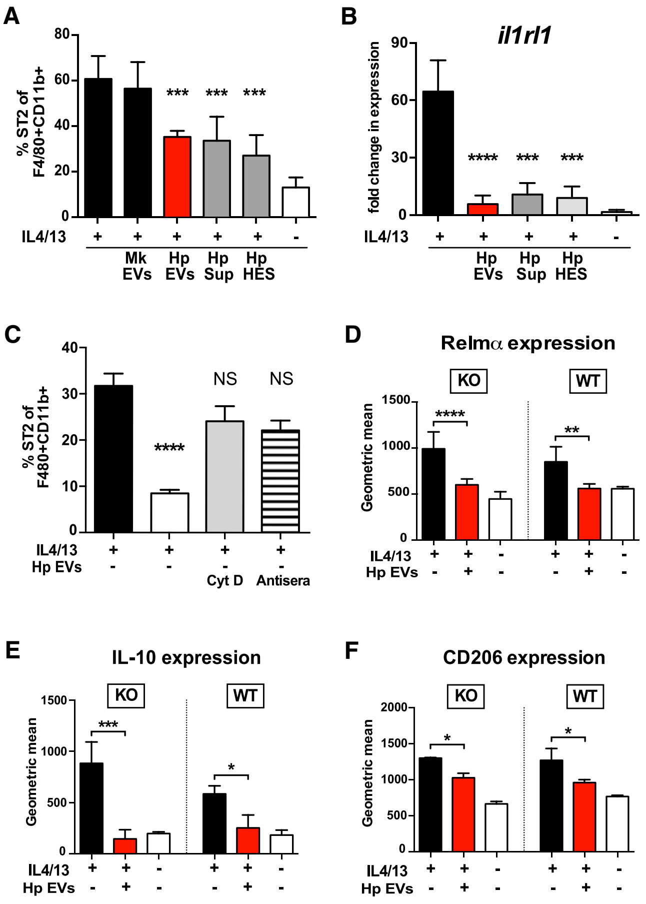

Figure 4. EVs Suppress ST2/IL-33R Expression in Macrophages during Type 2 Responses

(A and B) The percentage of (A) $ST2^+F4/80^+CD11b^+$ cells as determined by flow cytometry and transcriptional levels of (B) *Il1rl1* as determined by qRT-PCR analysis in BMDMs cultured with IL-4/IL-13 ± 5 μg/mL EVs, EV-depleted HES (sup), or total HES (HES) for 24 hr. MODE-K EVs (Mk exo) are used as a control. Gating strategy is shown in Figure S3A.

(C) The proportion of ST2-expressing BMDMs co-treated with 5 μg/mL EVs ± cytochalasin D or EV antisera, as determined by flow cytometry.

(D–F) Representative expression of (D) intracellular RELMα, (E) IL-10, or (F) surface CD206 in wild-type BALB/c (WT) and $T1/ST2^{-/-}$ (KO) BMDMs treated with 20 ng/mL IL-4/IL-13 ± 5 μg/mL EVs, as assessed by flow cytometry. Data are pooled from two to three independent experiments and presented as mean values ± SD (n = 6–12; one-way ANOVA, *p < 0.05, **p < 0.01, ***p < 0.001, and ****p < 0.0001; NS, not significant).

co-treatment with either cytochalasin D or polyclonal antisera (Figures 3F–3H). It is also prudent to mention that EV-elicited antiserum alone did not influence either alternative or classical activation in BMDMs (Figure S3H). Therefore, although antibody-mediated uptake of EVs was accelerated, it also directed EVs into a degradative lysosomal pathway (as is shown in Figures 2D and 2E), thereby ablating their functional effects.

EVs Suppress ST2/IL-33R Expression in Macrophages during Type 2 Responses

Alternative activation is also associated with expression of the ST2 subunit of the IL-33R. Notably, we previously demonstrated that *H. polygyrus* EVs suppress *il1rl1*, the gene for ST2, in MODE-K cells, and they modulate surface expression of ST2 on ILC2s in vivo (Buck et al., 2014). Thus, *H. polygyrus*-secreted EVs could be used to circumvent host danger signals through IL-33R that would normally lead to expulsion. We therefore tested ST2 surface protein expression in AAMϕ co-cultured with parasite-derived EVs, finding that EVs suppressed ST2 protein and mRNA (*il1rl1*) levels (Figures 4A and 4B; Figure S4A). Similar results were obtained with total HES or HES supernatant, suggesting further components in the secretion product can interfere with alternative activation. The effects of the EVs required their internalization, as EV-mediated suppression of ST2 on AAMϕ was abolished when cells were co-treated with either polyclonal EV antisera or cytochalasin D (Figure 4C).

To elucidate whether EV blockade of alternative activation is a consequence of ST2 inhibition, we isolated BMDMs from ST2-deficient mice (Rostan et al., 2015; Townsend et al., 2000). We found that EVs strongly suppressed the expression of intracellular RELMα and IL-10 and surface CD206 in AAMϕ from both wild-type BALB/c and ST2-deficient mice (Figures 4D–4F). A similar pattern emerged for the IL-4/IL-13-stimulated release of RELMα and Ym1 (Figures S4B and S4C), which were significantly repressed in both ST2-deficient and wild-type mice. This demonstrates that parasitic EVs can act independently of ST2 to suppress type 2 activation in macrophages.

EVs Stimulate Protective Immunity to *H. polygyrus* and Induce Specific Antibody Responses

As anti-EV antibodies blocked the immunomodulatory effects of EVs in vitro, we tested whether vaccination with EVs could engender protective immunity in mice in vivo, based on previous work showing antibody-dependent immunity in mice vaccinated with HES (Hewitson et al., 2011, 2015) (Figure 5A). Following immunization with EVs, HES, or HES supernatant in alum adjuvant (given at days 0, 28, and 35) and subsequent larval challenge, mice showed a marked reduction in fecal egg counts from day 14 to day 28 post-infection (Figure 5B). At day 28, intestinal worm burdens were reduced ~82% for EVs, ~98% for total HES, and ~78% for HES depleted of EVs (Figure 5C).

We then examined the antibody responses elicited by EV vaccination alone, prior to challenge infection (Figure S5A), which included IgM, IgG1, IgA, and IgE isotypes reactive with EVs (Figure 5D). Interestingly, mice immunized with HES or HES supernatant generated substantial levels of EV-responsive IgM, indicating a response to either shared molecular components or to cross-reactive epitopes, such as shared glycans (Hewitson et al., 2011). Likewise, sera from EV-vaccinated mice contained both IgM and IgG1 reactive to total HES and HES supernatant (Figures S5B and S5C).

The repertoire of antigens recognized by EV antiserum was then analyzed by western blot, showing differential binding to EV antigens compared to HES or EV-depleted HES proteins (Figure S5D). This is in line with previous mass spectrometry data highlighting specific enrichment of proteins in EVs (Buck et al., 2014). Hence, vaccination with EVs generated specific antibodies and was sufficient to interrupt infection by *H. polygyrus.*

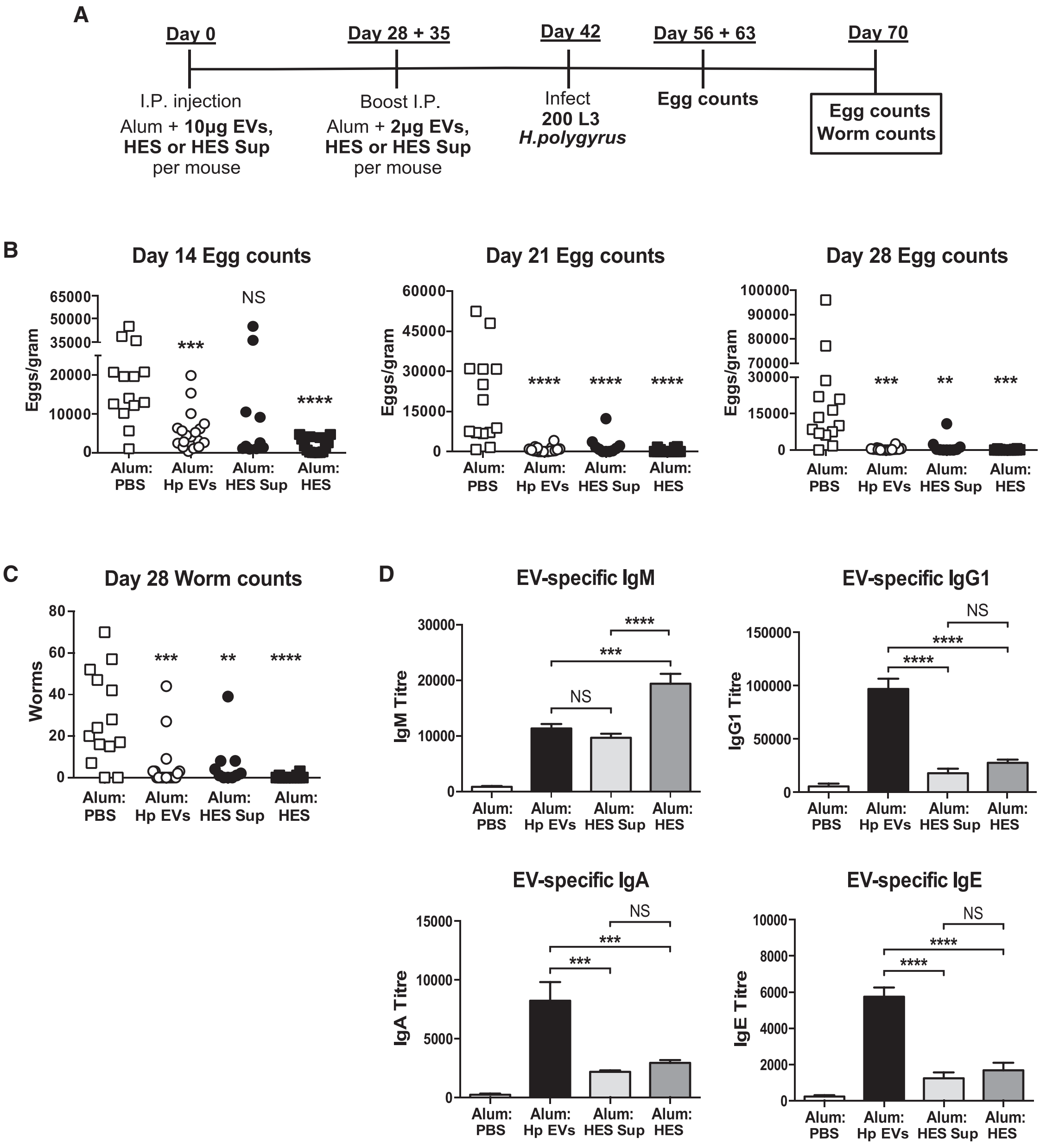

Figure 5. EVs Stimulate Protective Immunity against *H. polygyrus* Larval Challenge in C57BL/6 Mice, and They Induce Specific Antibody Responses In Vivo

(A) Age-matched female C57BL/6 mice were vaccinated with EVs, HES supernatant, total HES, or PBS in alum adjuvant, prior to challenge with 200 *H. polygyrus* L3 larvae.

(B) Egg counts per gram fecal matter were enumerated from *H. polygyrus*-challenged mice on days 14, 21, and 28.

(C) Adult worm counts from the small intestine on day 28 in *H. polygyrus*-challenged mice. Data are pooled from three experiments and presented as mean values ± SD (n = 10–18 mice per group; one-way ANOVA).

(D) Titers of IgM, IgG1, IgA, and IgE detectable from sera of immunized mice measured by ELISA coated with EVs. Data are representative of two independent experiments and presented as mean values ± SD (n = 5 mice per group; one-way ANOVA, *p < 0.05, **p < 0.01, ***p < 0.001, and ****p < 0.0001; NS, not significant).

ST2$^{-/-}$ Mice Are More Susceptible to *H. polygyrus* and Show Abrogated Alternative Activation of Macrophages

As previously mentioned, ST2-deficient mice are highly susceptible to helminth infection (Townsend et al., 2000), but surprisingly little data have been published on the course of *H. polygyrus* infection in this strain (Zaiss et al., 2013). We first demonstrated the susceptibility phenotype of the ST2$^{-/-}$ mouse during *H. polygyrus* infection, with higher egg counts and worm burdens than the partially resistant wild-type BALB/c genotype (Figures 6A and 6B). Consistent with previous data on

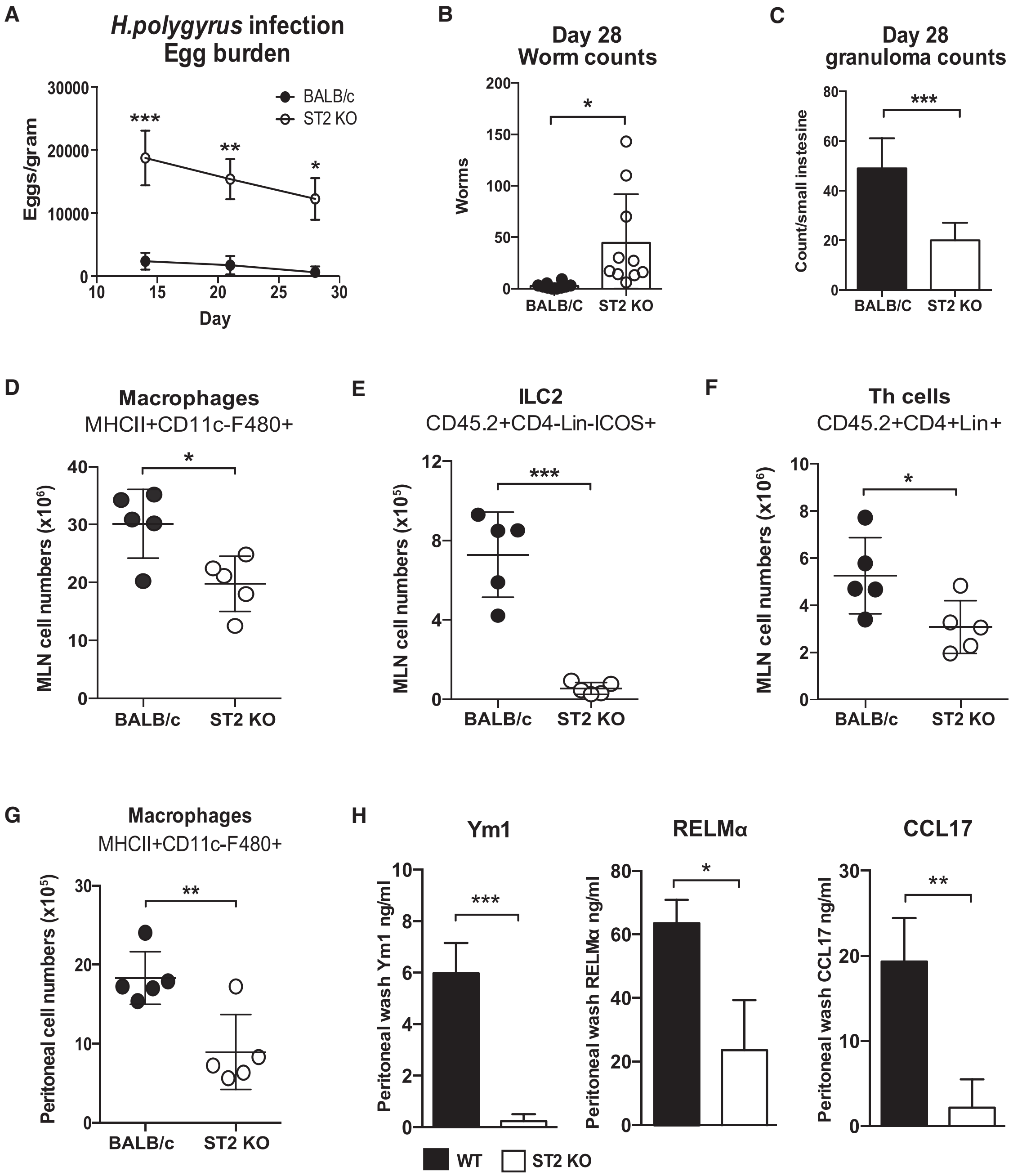

Figure 6. ST2-Deficient Mice Are Fully Susceptible to *H. polygyrus* Infection

(A) Female T1/ST2$^{-/-}$ and wild-type BALB/c mice were infected with 200 *H. polygyrus* L3 larvae, and egg counts per gram fecal matter were enumerated on days 14, 21, and 28 post-infection.

(B and C) Intestinal adult (B) worm burden and (C) granulomas were enumerated on day 28.

(D–F) MLNs recovered from *H. polygyrus*-infected BALB/c and T1/ST2$^{-/-}$ mice were isolated at day 28 post-infection, and absolute numbers of (D) macrophages (F4/80^{+}CD11c^{-}MHCII^{+}), (E) ILC2s (Lineage^{-}CD4^{-}ICOS^{+}), and (F) T helper cells (CD4^{+}Lineage^{+}) were determined by flow cytometry.

(G and H) Absolute numbers of (G) macrophages isolated from the peritoneal lavage (gated as in D) were quantified by flow cytometry, and levels of myeloid-derived cytokines (H) Ym1, RELMα, and CCL17 were assessed by ELISA. (A and B) Data are pooled from two independent experiments (n = 10); (C–H) data are representative from one of two experiments and presented as mean values ± SD (n = 5 mice per group; Student's t test, *$p < 0.05$, **$p < 0.01$, and ***$p < 0.001$; NS, not significant).

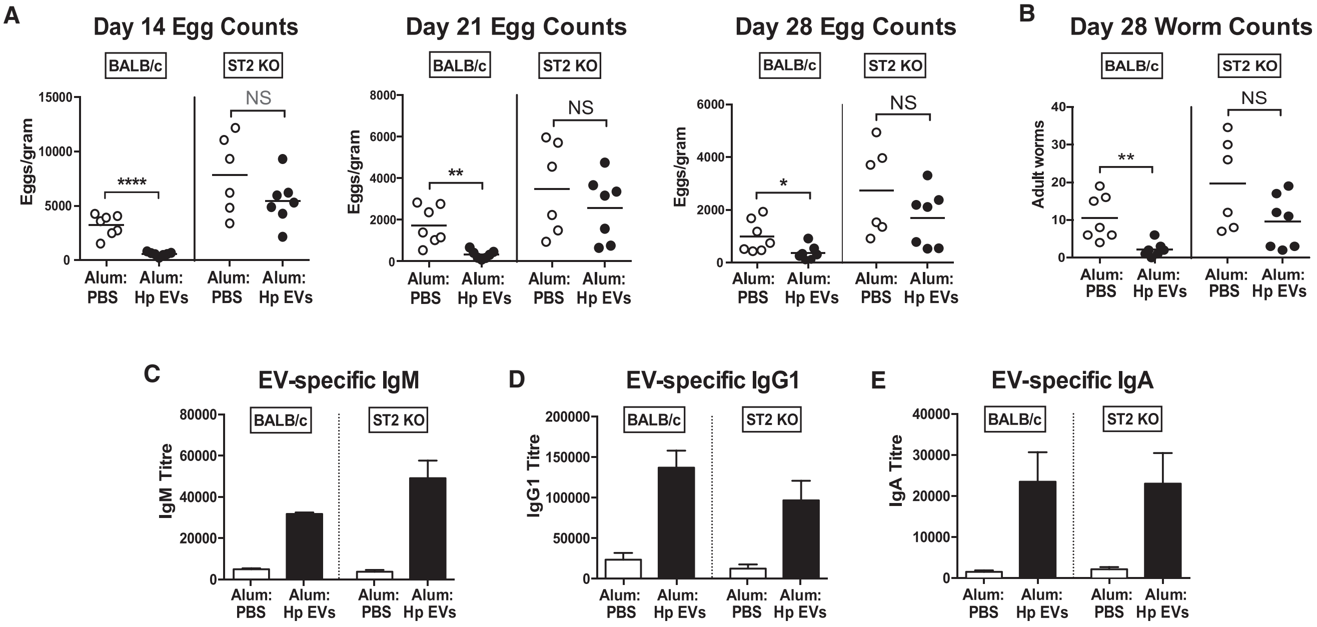

Figure 7. Vaccination against EVs Is Poorly Protective in *H. polygyrus*-Susceptible ST2-Deficient Mice

(A) Female T1/ST2$^{-/-}$ and BALB/c mice were vaccinated with EVs or PBS in alum adjuvant prior to challenge with 200 *H. polygyrus* L3 larvae (as Figure 5A), and eggs counts per gram fecal matter were enumerated on days 14, 21, and 28 post-infection.

(B) Intestinal adult worm burdens were measured on day 28.

(C–E) EV-specific titers of (C) IgM, (D) IgG1, and (E) IgA detectable from sera of EV- or PBS-immunized mice measured by ELISA. (A–E) Data are pooled from two independent experiments (n = 6–7), presented as mean values ± SD (Student's t test, *p < 0.05, **p < 0.01, and ****p < 0.0001; NS, not significant).

S. mansoni-infected ST2$^{-/-}$ mice (Townsend et al., 2000), there were significantly fewer granulomas in the small intestine of infected ST2$^{-/-}$ mice (Figure 6C). Although there were similar total cell numbers in the mesenteric lymph nodes (MLNs) of both wild-type and ST2$^{-/-}$ mice (Figure S6A), there were significantly lower numbers of macrophages, ILC2s, and CD4^{+} T cells in ST2$^{-/-}$ mice (Figures 6D–6F), whereas the T regulatory cell population was unaffected (Figure S6B). The decrease in ILC2s from ST2$^{-/-}$ mice was not unexpected, as they show lower levels of ILC2s in the lung during allergic airway inflammation (Flaczyk et al., 2013) and *N. brasiliensis* infection (Bando et al., 2013). In the peritoneum, as in the MLN, there were significantly lower numbers of macrophages in ST2$^{-/-}$ mice (Figure 6G), and, most significantly, expression of the AAMΦ-associated proteins Ym1, RELMα, and CCL17 was greatly attenuated in the animals lacking ST2 (Figure 6H).

Vaccination against EVs Provides Inadequate Protection in *H. polygyrus*-Susceptible ST2-Deficient Mice

We next wanted to test whether immunity generated by EV vaccination was compromised in ST2$^{-/-}$ mice, which lack one of the signaling pathways targeted by EVs. We therefore vaccinated either BALB/c or ST2$^{-/-}$ mice against *H. polygyrus* EVs before challenge with infective third-stage larvae, and we monitored the course of infection for 28 days. As expected, wild-type BALB/c mice vaccinated with EV-alum had potent immunity to helminth infection, with markedly reduced egg counts and worm burdens (Figures 7A and 7B). In contrast, ST2$^{-/-}$ mice harbored higher worm numbers that were not significantly altered by vaccination, although there was a trend toward reduced egg numbers and a gradual loss of parasites over time. Finally, we looked at the repertoire (Figure S7A) and titer (Figures 7C–7E; Figure S7B) of EV-specific serum antibodies generated in vaccinated mice post-infection. Notably, both ST2$^{-/-}$ and wild-type mice generated comparable levels of IgM, IgG1, and IgA responsive to EVs, arguing that susceptibility in the absence of IL-33 signaling is due to a deficiency in a cellular, rather than a humoral, component of the immune response.

DISCUSSION

The ability of parasitic helminths, such as *H. polygyrus*, to release EVs identifies a newly recognized vehicle to mediate cross-species communication as part of the host-parasite relationship (Coakley et al., 2015). However, the rapidly expanding body of data demonstrating that EVs are secreted by diverse parasites raises key questions of how they function in host cells and the specificity of the cell populations with which they interact.

Here we have shown that macrophages efficiently take up *H. polygyrus*-derived EVs, implicating a phagocytic or endocytic pathway that can be blocked by cytochalasin D and enhanced by specific antibodies, presumably through opsonization. After a 1-hr incubation, *H. polygyrus* EVs localized discretely within BMDMs (Figure 1B), and they became dispersed after 8 hr. It could, therefore, be hypothesized that, in the early events of uptake, parasitic EVs are still within endosomes, reminiscent of previous observations demonstrating similar intracellular

endosomal aggregates shortly after EV internalization (Morelli et al., 2004; Tian et al., 2010).

While uptake by macrophages might initially be interpreted as a host mechanism for the removal of parasite EVs, we show that the nematode-derived EVs exert functional properties on the recipient macrophages. Indeed, EVs generate potent suppression of type 1 and type 2 effector molecules when administered during or after the onset of macrophage activation. Among the molecules suppressed, arginase-1 is an essential component of protective immunity to intestinal helminths (Anthony et al., 2006; Obata-Ninomiya et al., 2013). We do not yet know which parasite molecule(s) within the EV cargo is responsible for these effects, as there is a suite of protein, lipid, and RNA candidates that merit further study (Buck et al., 2014; Simbari et al., 2016). However, *S. mansoni* ES can be internalized following CD206 binding on macrophages, leading to diminished Th2 cell responses and chronic infection (Paveley et al., 2011), an avenue that may also be exploited by *H. polygyrus* EVs. Importantly, treatments that modulate EV uptake in AAMϕ, such as cytochalasin D or EV antibodies, abrogated EV-suppressive effects. In the case of EV antibodies, intracellular imaging data suggest that they alter the intracellular localization of EVs, directing them into the lysosomal pathway.

EVs induce high titers of IgM, IgG1, and IgA serum antibodies in mice vaccinated with EV-alum adjuvant. Strong EV-responsive IgG1 antibody responses could be important for downstream resistance during *H. polygyrus* infection; antibodies against total HES were essential for immunity to larval challenge, limiting migration of the parasite from the sub-mucosa (Hewitson et al., 2011, 2015). Vaccination with EVs generated similar immunity, based on both eggs counts and subsequent worm burden. As there is comparable protection among HES, EVs, or HES supernatant, immunogenic epitopes may be shared among all preparations, since there are some shared proteins among these (Buck et al., 2014). It will be interesting to identify EV-specific antigens that raise antibody responses, as they may provide a future vaccine candidate against nematode infection. Interestingly, a recent study demonstrates that immunizing against *Echinostoma caproni* EVs can reduce symptom severity during infection, suggesting that parasitic EVs may hold therapeutic potential in a wide range of helminth infections (Trelis et al., 2016).

Both wild-type and $ST2^{-/-}$ BMDMs stimulated via the IL-4Rα pathway exhibited similar EV-mediated suppression of alternative activation, suggesting that the impact of EVs on alternative activation is not via ST2. In support of this, a recent study showed that disruption of ST2 signaling had no effect on type 2-mediated responses, including IL-4 release, following challenge by *S. mansoni* (Vannella et al., 2016). However, during infection with *H. polygyrus*, we recovered a lower proportion of macrophages and AAMϕ-associated proteins in $ST2^{-/-}$ mice. Therefore, the effects of ST2 deficiency on type 2 immunity may be context or model dependent. As ILC2s are a potent source of type 2 cytokines, we hypothesize that their low numbers in $ST2^{-/-}$ mice contribute to the reduced alternative activation of macrophages that we observed. Furthermore, as macrophages are key players in mediating immunity to *H. polygyrus* (Reynolds et al., 2012), this may explain the susceptibility of $ST2^{-/-}$ mice to infection. Unlike the near-sterile immunity induced in wild-type mice, there was no significant protection induced by EV-alum vaccination in parasite-susceptible $ST2^{-/-}$ mice, thus demonstrating the essential requirement for IL-33 signaling in resistance to this parasite. Notably, ST2-deficient mice mount equivalent antibody responses, yet they are unable to clear the parasite; hence, even in the presence of antibody, an IL-33-dependent cell population is necessary for effective immunity. Equally, the importance of IL-33 signaling explains why the parasite targets this pathway, blockading both the ligand (McSorley et al., 2014) and, as we show here, the receptor. Defining the molecular mechanisms that occur during IL-33R-EV interactions may be a future objective for therapies to drive innate immunity and treat parasitic infection. It will be particularly interesting to determine whether EVs secreted by other parasites modulate the IL-33/ST2 axis to promote susceptibility (Rostan et al., 2015). Given recent data showing that total ES from the liver fluke *Fasciola hepatica* actually enhanced ST2 expression on peritoneal CD4+ T helper cells, there may be contrasting mechanisms by which different helminths interact with the IL-33 pathway (Finlay et al., 2016).

In conclusion, these results demonstrate the ability of helminth EVs to modulate both alternative activation and IL-33-mediated signaling, solidifying them as critical parasite virulence factors. Furthermore, targeting EVs by vaccination provided protective immunity against the parasite, highlighting their key role in establishing infection. Given their biological complexity, a deeper understanding of the properties of parasitic EVs will be key to determining how they efficiently mediate cross-species communication. Such insights will be crucial to determine how we can interfere with or mimic these processes to treat infectious and inflammatory diseases.

EXPERIMENTAL PROCEDURES

Mice

Female C57BL/6 mice, BALB/c mice (6–10 weeks old), and $T1/ST2^{-/-}$ ($ST2^{-/-}$) mice (kindly provided by Dr. Andrew McKenzie, MRC Laboratory of Molecular Biology [LMB]) were used for immunization studies and culture of bone marrow-derived myeloid populations. Male CBA × C57BL/6 F1 (CBF1) mice were used to maintain the *H. polygyrus* life cycle. All mice were bred in house, and animal studies were performed under UK Home Office Licenses with institutional oversight provided by qualified veterinarians.

EV Isolation

To obtain HES products, CBF1 mice were infected with L3 stage larvae by oral gavage, with adult parasites collected from the small intestine 14 days post-infection, as described previously (Johnston et al., 2015). Following isolation, adult *H. polygyrus* worms were kept in serum-free media in vitro, and the secretion product was collected every 3 days for 14 days. Eggs were removed by spinning at 400 × *g* before filtering through a 0.2-μm filter (Millipore). Purified medium was then spun at 100,000 × *g* for 2 hr in polyallomer tubes at 4°C in a SW40 rotor (Beckman Coulter). The ultracentrifuged pellet was washed twice in filtered PBS at 100,000 × *g* for 2 hr, and the final pellet was resuspended in PBS and protein content was quantified by Qubit (Invitrogen) and stored at −80°C. Qualitative analysis of EV purity was carried out using silver stain analysis as described previously (Buck et al., 2014). The resulting supernatant (and total HES product) was concentrated using Vivaspin 6 5000 MWCO tubes (Fisher) at 5,000 × *g*, and it was washed twice with PBS.

Cell Culture

Bone marrow stem cells were flushed from femurs and tibiae of 6- to 10-week-old female C57BL/6, BALB/c, and $ST2^{-/-}$ mice. Bone marrow macrophages were generated by incubating cells at 37°C in 5% CO_2 in complete DMEM

(Invitrogen), supplemented with 20% fetal bovine serum (Invitrogen), 1% penicillin/streptomycin (Lonza), 1% glutamine (Lonza), and conditioned medium from L929 cells (containing macrophage colony stimulating factor [M-CSF]) for 7 days. For cell stimulation experiments, BMDMs were incubated with 20 ng/mL IL-4/IL-13 or 500 ng/mL LPS for 24–48 hr. MODE-K cells were kindly provided by Dominique Kaiserlian (INSERM) and grown as previously described (Vidal et al., 1993). RAW264.7 cells were cultured in RPMI-1640 (Sigma) supplemented with 10% fetal bovine serum, 1% penicillin/streptomycin, and 1% L-glutamine.

Uptake Assays

EVs purified from *H. polygyrus*, MODE-K, or RAW264.7 cells were labeled with PKH67 or CellVue Claret dye (Sigma) for 5 min at room temperature, according to the manufacturer's instructions; 2 μg dye was used per 5 μg EVs. The staining reaction was stopped by adding an equal amount of 1% purified BSA, and EVs were washed in PBS by ultracentrifugation (1 hr at 100,000 × *g*). A control solution was prepared with PKH67/CellVue in PBS in the absence of EVs. Experiments were carried out with 2.5–5 μg EVs per 200,000 cells for varying time points at 37°C. Cells were then washed twice in PBS before analysis by flow cytometry or confocal microscopy. In some experiments, control samples were treated similarly before the addition of 50 μl 0.25% trypsin/EDTA (Gibco) for 5 min in order to remove EVs that may have remained on the cell surface.

Confocal Microscopy

BMDMs were allowed to attach to coverslips overnight, and the following day they were transferred to complete DMEM supplemented with 1% L-glutamine. Cells were incubated with labeled EVs or controls before subsequent staining in 1:300 F4/80-AF647 in PBS. Cells were then fixed with 4% paraformaldehyde (PFA), with residual PFA quenched with 50 mM glycine. The coverslips were washed four times in PBS, and nuclei were stained with DAPI-supplemented ProLong Fade Gold (Invitrogen) mounting media. Samples were examined on a Leica SP5 II microscope (Leica Microsystems, lasers exciting at 405 and 488 nm, 63× objective) using LAS AP software (Leica).

Imagestream

BMDMs were incubated for 24 hr with CellVue-labeled *H. polygyrus*- or RAW264.7-derived EVs, and then they were stained with 50 nM LysoTracker Green DND-26 (Cell Signaling Technology) for 60 min at 37°C. At least 10,000 live-cell images for each sample were acquired using a six-channel ImageStream^{X} Mark II (EMD Millipore) imaging flow cytometer equipped with 405-, 488-, and 642-nm lasers. Samples and single-color compensation controls were acquired at 40× magnification. Data were analyzed using IDEAS 4.0.735 software (EMD Millipore) for Lysotracker/CellVue Bright Detail Similarity (BDS). BDS is a normalized measurement of co-localization or overlap between the fluorescent signals and pixel intensity, as described elsewhere (Phadwal et al., 2012).

Enzyme-Linked Immunosorbent Assay

Levels of macrophage proteins were measured by ELISA for IL-6, IL-12p40, and TNF (all eBioscience); RELMα (PeproTech); and CCL17 or Chitinase 3-like protein 1 (Ym1) (both R&D Systems), according to the manufacturer's instructions. Plates were developed with streptavidin-alkaline phosphatase and p-nitrophenyl phosphate substrate (both Sigma), and they were read at 405 nm. In serum antibody ELISAs, blood was collected via cardiac puncture, clotted for 1 hr at room temperature, and then spun for 20 min at 2,500 × *g* to remove red blood cells (RBCs). After blocking at 37°C with 10% BSA in carbonate buffer, sera were added in serial dilutions to ELISA plates coated with either 1 μg/mL HES, EV-depleted HES, or EVs. Antibody binding was detected using horseradish peroxidase (HRP)-conjugated goat anti-mouse IgM, IgG1, IgA, or IgE (Southern Biotech) and ABTS Peroxidase Substrate (KPL), and it was read at 405 nm.

Flow Cytometry

Single-cell suspensions were made from bone marrow myeloid populations, MLN, or peritoneal washes, and subsequently they were washed in PBS containing 0.5% BSA (Sigma). Live/dead Fixable Aqua dye (Invitrogen) was used to exclude dead cells. The following antibodies (Abs) were used: lineage markers (anti-CD3/CD4/CD5/CD19/CD11b/CD11c/CD19/GR1), ILC2 markers (ICOS/ST2), T regulatory cells (CD4/CD25/Foxp3), and macrophages (F480/CD11b/MHCII/ST2/CD206), including all appropriate isotype controls. For intracellular cytokine staining, BMDMs were stained as above and then permeabilized with the BD Biosciences Fixation/Permeabilization kit (as per the manufacturer's instructions), before staining with IL-10 or RELMα antibodies. Samples were acquired on a Becton Dickinson LSRII flow cytometer and data were analyzed using FlowJo software (Tree Star).

Reverse Transcription and Real-Time PCR for mRNA

Reverse transcription reactions were performed using miScript System kit II (QIAGEN) according to the manufacturer's instructions. Real-time SYBR-green PCR assays for mRNA detection were performed using Light Cycler System (Roche). For primers (Invitrogen), see the Supplemental Experimental Procedures. Data were analyzed using the standard 2 Δ*C*t method, and targets were normalized to the housekeeping gene GAPDH.

Vaccination

For the generation of polyclonal antibodies against EVs, rats were immunized with 75 μg EVs in alum adjuvant intraperitoneally (i.p.), and then they were boosted with 15 μg EVs in alum adjuvant on days 28 and 35, before serum collection on day 42. Mice were immunized with 10 μg EVs, HES, or HES depleted of EVs in alum adjuvant (i.p.), and then they were boosted on days 28 and 35 with 2 μg of the same immunogen in alum (i.p.). In some experiments, mice were challenged with 200 *H. polygyrus* L3 larvae on day 42, with fecal egg and adult worm counts up to 28 days later. Egg and worm numbers were determined as eggs per gram fecal matter and total adult worms in the small intestine.

Statistical Analysis

Data were analyzed using Prism 6 (GraphPad). In-group variance was assessed by Brown Forsythe test, and data were log-transformed and analyzed by one- or two-way ANOVA, with a Tukey's multiple comparisons post-test (****$p < 0.0001$, ***$p < 0.001$, **$p < 0.01$, and *$p < 0.05$; NS, not significant, $p > 0.05$).

SUPPLEMENTAL INFORMATION

Supplemental Information includes Supplemental Experimental Procedures and seven figures and can be found with this article online at http://dx.doi.org/10.1016/j.celrep.2017.05.001.

AUTHOR CONTRIBUTIONS

G.C. co-designed and performed all experiments and subsequent analysis and wrote the paper. J.L.M. co-designed and supported macrophage functional assays/vaccination studies. J.G.B. and F.S. co-designed and supported EV uptake assays. E.R., M.M., and Y.H. contributed to vaccination studies and maintenance of the *H. polygyrus* life cycle. H.J.M. contributed to discussions and interpretation of T1/ST2 knockout mouse data. A.H.B. and R.M.M. supervised and co-designed experiments, interpreted results, and edited the manuscript.

ACKNOWLEDGMENTS

This work was supported by a studentship to G.C. from the Biotechnology and Biological Sciences Research Council (BB/J01446X/1), as well as by a fellowship (AHB-097394) and an investigator award (RMM-106122) from the Wellcome Trust, a Wellcome Trust strategic award to the Centre for Immunity, Infection & Evolution at the University of Edinburgh (095831), and core funding from the Wellcome Trust for the Wellcome Centre for Molecular Parasitology (Ref 104111). In addition, we would like to thank Katrina Gordon for many helpful discussions and Martin Waterfall for technical support with flow cytometry.

Received: October 27, 2016
Revised: March 30, 2017
Accepted: April 27, 2017
Published: May 23, 2017

REFERENCES

Anthony, R.M., Urban, J.F., Jr., Alem, F., Hamed, H.A., Rozo, C.T., Boucher, J.L., Van Rooijen, N., and Gause, W.C. (2006). Memory T(H)2 cells induce alternatively activated macrophages to mediate protection against nematode parasites. Nat. Med. *12*, 955–960.

Bando, J.K., Nussbaum, J.C., Liang, H.E., and Locksley, R.M. (2013). Type 2 innate lymphoid cells constitutively express arginase-I in the naive and inflamed lung. J. Leukoc. Biol. *94*, 877–884.

Behnke, J.M., Menge, D.M., and Noyes, H. (2009). *Heligmosomoides bakeri*: a model for exploring the biology and genetics of resistance to chronic gastrointestinal nematode infections. Parasitology *136*, 1565–1580.

Buck, A.H., Coakley, G., Simbari, F., McSorley, H.J., Quintana, J.F., Le Bihan, T., Kumar, S., Abreu-Goodger, C., Lear, M., Harcus, Y., et al. (2014). Exosomes secreted by nematode parasites transfer small RNAs to mammalian cells and modulate innate immunity. Nat. Commun. *5*, 5488.

Chaiyadet, S., Smout, M., Johnson, M., Whitchurch, C., Turnbull, L., Kaewkes, S., Sotillo, J., Loukas, A., and Sripa, B. (2015). Excretory/secretory products of the carcinogenic liver fluke are endocytosed by human cholangiocytes and drive cell proliferation and IL6 production. Int. J. Parasitol. *45*, 773–781.

Coakley, G., Maizels, R.M., and Buck, A.H. (2015). Exosomes and other extracellular vesicles: The new communicators in parasite infections. Trends Parasitol. *31*, 477–489.

Coakley, G., Buck, A.H., and Maizels, R.M. (2016). Host parasite communications-Messages from helminths for the immune system: Parasite communication and cell-cell interactions. Mol. Biochem. Parasitol. *208*, 33–40.

Cwiklinski, K., de la Torre-Escudero, E., Trelis, M., Bernal, D., Dufresne, P.J., Brennan, G.P., O'Neill, S., Tort, J., Paterson, S., Marcilla, A., et al. (2015). The Extracellular Vesicles of the Helminth Pathogen, Fasciola hepatica: Biogenesis Pathways and Cargo Molecules Involved in Parasite Pathogenesis. Mol. Cell. Proteomics *14*, 3258–3273.

Deatherage, B.L., and Cookson, B.T. (2012). Membrane vesicle release in bacteria, eukaryotes, and archaea: a conserved yet underappreciated aspect of microbial life. Infect. Immun. *80*, 1948–1957.

Escrevente, C., Keller, S., Altevogt, P., and Costa, J. (2011). Interaction and uptake of exosomes by ovarian cancer cells. BMC Cancer *11*, 108.

Feng, X., Deng, T., Zhang, Y., Su, S., Wei, C., and Han, D. (2011). Lipopolysaccharide inhibits macrophage phagocytosis of apoptotic neutrophils by regulating the production of tumour necrosis factor α and growth arrest-specific gene 6. Immunology *132*, 287–295.

Filbey, K.J., Grainger, J.R., Smith, K.A., Boon, L., van Rooijen, N., Harcus, Y., Jenkins, S., Hewitson, J.P., and Maizels, R.M. (2014). Innate and adaptive type 2 immune cell responses in genetically controlled resistance to intestinal helminth infection. Immunol. Cell Biol. *92*, 436–448.

Finlay, C.M., Stefanska, A.M., Walsh, K.P., Kelly, P.J., Boon, L., Lavelle, E.C., Walsh, P.T., and Mills, K.H. (2016). Helminth Products Protect against Autoimmunity via Innate Type 2 Cytokines IL-5 and IL-33, Which Promote Eosinophilia. J. Immunol. *196*, 703–714.

Flaczyk, A., Duerr, C.U., Shourian, M., Lafferty, E.I., Fritz, J.H., and Qureshi, S.T. (2013). IL-33 signaling regulates innate and adaptive immunity to *Cryptococcus neoformans*. J. Immunol. *191*, 2503–2513.

Gause, W.C., Wynn, T.A., and Allen, J.E. (2013). Type 2 immunity and wound healing: evolutionary refinement of adaptive immunity by helminths. Nat. Rev. Immunol. *13*, 607–614.

Gonçalves, M.F., Umezawa, E.S., Katzin, A.M., de Souza, W., Alves, M.J., Zingales, B., and Colli, W. (1991). *Trypanosoma cruzi*: shedding of surface antigens as membrane vesicles. Exp. Parasitol. *72*, 43–53.

Hansen, E.P., Kringel, H., Williams, A.R., and Nejsum, P. (2015). Secretion of RNA-containing extracellular vesicles by the porcine whipworm, *Trichuris suis*. J. Parasitol. *101*, 336–340.

Heusermann, W., Hean, J., Trojer, D., Steib, E., von Bueren, S., Graff-Meyer, A., Genoud, C., Martin, K., Pizzato, N., Voshol, J., et al. (2016). Exosomes surf on filopodia to enter cells at endocytic hot spots, traffic within endosomes, and are targeted to the ER. J. Cell Biol. *213*, 173–184.

Hewitson, J.P., Filbey, K.J., Grainger, J.R., Dowle, A.A., Pearson, M., Murray, J., Harcus, Y., and Maizels, R.M. (2011). *Heligmosomoides polygyrus* elicits a dominant nonprotective antibody response directed against restricted glycan and peptide epitopes. J. Immunol. *187*, 4764–4777.

Hewitson, J.P., Filbey, K.J., Esser-von Bieren, J., Camberis, M., Schwartz, C., Murray, J., Reynolds, L.A., Blair, N., Robertson, E., Harcus, Y., et al. (2015). Concerted activity of IgG1 antibodies and IL-4/IL-25-dependent effector cells trap helminth larvae in the tissues following vaccination with defined secreted antigens, providing sterile immunity to challenge infection. PLoS Pathog. *11*, e1004676.

Hoppstädter, J., Seif, M., Dembek, A., Cavelius, C., Huwer, H., Kraegeloh, A., and Kiemer, A.K. (2015). M2 polarization enhances silica nanoparticle uptake by macrophages. Front. Pharmacol. *6*, 55.

Johnston, C.J., Robertson, E., Harcus, Y., Grainger, J.R., Coakley, G., Smyth, D.J., McSorley, H.J., and Maizels, R. (2015). Cultivation of *Heligmosomoides polygyrus*: an immunomodulatory nematode parasite and its secreted products. J. Vis. Exp. (*98*), e52412.

Kreider, T., Anthony, R.M., Urban, J.F., Jr., and Gause, W.C. (2007). Alternatively activated macrophages in helminth infections. Curr. Opin. Immunol. *19*, 448–453.

Kurowska-Stolarska, M., Stolarski, B., Kewin, P., Murphy, G., Corrigan, C.J., Ying, S., Pitman, N., Mirchandani, A., Rana, B., van Rooijen, N., et al. (2009). IL-33 amplifies the polarization of alternatively activated macrophages that contribute to airway inflammation. J. Immunol. *183*, 6469–6477.

Kusuma, R.J., Manca, S., Friemel, T., Sukreet, S., Nguyen, C., and Zempleni, J. (2016). Human vascular endothelial cells transport foreign exosomes from cow's milk by endocytosis. Am. J. Physiol. Cell Physiol. *310*, C800–C807.

Marcilla, A., Trelis, M., Cortés, A., Sotillo, J., Cantalapiedra, F., Minguez, M.T., Valero, M.L., Sánchez del Pino, M.M., Muñoz-Antoli, C., Toledo, R., and Bernal, D. (2012). Extracellular vesicles from parasitic helminths contain specific excretory/secretory proteins and are internalized in intestinal host cells. PLoS ONE *7*, e45974.

McSorley, H.J., Blair, N.F., Smith, K.A., McKenzie, A.N., and Maizels, R.M. (2014). Blockade of IL-33 release and suppression of type 2 innate lymphoid cell responses by helminth secreted products in airway allergy. Mucosal Immunol. *7*, 1068–1078.

Molofsky, A.B., Savage, A.K., and Locksley, R.M. (2015). Interleukin-33 in Tissue homeostasis, injury, and inflammation. Immunity *42*, 1005–1019.

Morelli, A.E., Larregina, A.T., Shufesky, W.J., Sullivan, M.L., Stolz, D.B., Papworth, G.D., Zahorchak, A.F., Logar, A.J., Wang, Z., Watkins, S.C., et al. (2004). Endocytosis, intracellular sorting, and processing of exosomes by dendritic cells. Blood *104*, 3257–3266.

Neill, D.R., Wong, S.H., Bellosi, A., Flynn, R.J., Daly, M., Langford, T.K., Bucks, C., Kane, C.M., Fallon, P.G., Pannell, R., et al. (2010). Nuocytes represent a new innate effector leukocyte that mediates type-2 immunity. Nature *464*, 1367–1370.

Nowacki, F.C., Swain, M.T., Klychnikov, O.I., Niazi, U., Ivens, A., Quintana, J.F., Hensbergen, P.J., Hokke, C.H., Buck, A.H., and Hoffmann, K.F. (2015). Protein and small non-coding RNA-enriched extracellular vesicles are released by the pathogenic blood fluke *Schistosoma mansoni*. J. Extracell. Vesicles *4*, 28665.

Obata-Ninomiya, K., Ishiwata, K., Tsutsui, H., Nei, Y., Yoshikawa, S., Kawano, Y., Minegishi, Y., Ohta, N., Watanabe, N., Kanuka, H., and Karasuyama, H. (2013). The skin is an important bulwark of acquired immunity against intestinal helminths. J. Exp. Med. *210*, 2583–2595.

Paveley, R.A., Aynsley, S.A., Turner, J.D., Bourke, C.D., Jenkins, S.J., Cook, P.C., Martinez-Pomares, L., and Mountford, A.P. (2011). The Mannose Receptor (CD206) is an important pattern recognition receptor (PRR) in the detection of the infective stage of the helminth *Schistosoma mansoni* and modulates IFNγ production. Int. J. Parasitol. *41*, 1335–1345.

Pearson, M.S., Tribolet, L., Cantacessi, C., Periago, M.V., Valero, M.A., Jariwala, A.R., Hotez, P., Diemert, D., Loukas, A., and Bethony, J. (2012). Molecular mechanisms of hookworm disease: stealth, virulence, and vaccines. J. Allergy Clin. Immunol. *130*, 13–21.

Perrigoue, J.G., Marshall, F.A., and Artis, D. (2008). On the hunt for helminths: innate immune cells in the recognition and response to helminth parasites. Cell. Microbiol. *10*, 1757–1764.

Phadwal, K., Alegre-Abarrategui, J., Watson, A.S., Pike, L., Anbalagan, S., Hammond, E.M., Wade-Martins, R., McMichael, A., Klenerman, P., and Simon, A.K. (2012). A novel method for autophagy detection in primary cells: impaired levels of macroautophagy in immunosenescent T cells. Autophagy *8*, 677–689.

Reynolds, L.A., Filbey, K.J., and Maizels, R.M. (2012). Immunity to the model intestinal helminth parasite *Heligmosomoides polygyrus*. Semin. Immunopathol. *34*, 829–846.

Rostan, O., Arshad, M.I., Piquet-Pellorce, C., Robert-Gangneux, F., Gangneux, J.P., and Samson, M. (2015). Crucial and diverse role of the interleukin-33/ST2 axis in infectious diseases. Infect. Immun. *83*, 1738–1748.

Rückerl, D., and Allen, J.E. (2014). Macrophage proliferation, provenance, and plasticity in macroparasite infection. Immunol. Rev. *262*, 113–133.

Scalfone, L.K., Nel, H.J., Gagliardo, L.F., Cameron, J.L., Al-Shokri, S., Leifer, C.A., Fallon, P.G., and Appleton, J.A. (2013). Participation of MyD88 and interleukin-33 as innate drivers of Th2 immunity to *Trichinella spiralis*. Infect. Immun. *81*, 1354–1363.

Silverman, J.M., Clos, J., Horakova, E., Wang, A.Y., Wiesgigl, M., Kelly, I., Lynn, M.A., McMaster, W.R., Foster, L.J., Levings, M.K., and Reiner, N.E. (2010). *Leishmania* exosomes modulate innate and adaptive immune responses through effects on monocytes and dendritic cells. J. Immunol. *185*, 5011–5022.

Simbari, F., McCaskill, J., Coakley, G., Millar, M., Maizels, R.M., Fabriás, G., Casas, J., and Buck, A.H. (2016). Plasmalogen enrichment in exosomes secreted by a nematode parasite versus those derived from its mouse host: implications for exosome stability and biology. J. Extracell. Vesicles *5*, 30741.

Szempruch, A.J., Sykes, S.E., Kieft, R., Dennison, L., Becker, A.C., Gartrell, A., Martin, W.J., Nakayasu, E.S., Almeida, I.C., Hajduk, S.L., and Harrington, J.M. (2016). Extracellular vesicles from *Trypanosoma brucei* mediate virulence factor transfer and cause host anemia. Cell *164*, 246–257.

Tian, T., Wang, Y., Wang, H., Zhu, Z., and Xiao, Z. (2010). Visualizing of the cellular uptake and intracellular trafficking of exosomes by live-cell microscopy. J. Cell. Biochem. *111*, 488–496.

Townsend, M.J., Fallon, P.G., Matthews, D.J., Jolin, H.E., and McKenzie, A.N. (2000). T1/ST2-deficient mice demonstrate the importance of T1/ST2 in developing primary T helper cell type 2 responses. J. Exp. Med. *191*, 1069–1076.

Trelis, M., Galiano, A., Bolado, A., Toledo, R., Marcilla, A., and Bernal, D. (2016). Subcutaneous injection of exosomes reduces symptom severity and mortality induced by *Echinostoma caproni* infection in BALB/c mice. Int. J. Parasitol. *46*, 799–808.

Twu, O., de Miguel, N., Lustig, G., Stevens, G.C., Vashisht, A.A., Wohlschlegel, J.A., and Johnson, P.J. (2013). Trichomonas vaginalis exosomes deliver cargo to host cells and mediate host:parasite interactions. PLoS Pathog. *9*, e1003482.

Tzelos, T., Matthews, J.B., Buck, A.H., Simbari, F., Frew, D., Inglis, N.F., McLean, K., Nisbet, A.J., Whitelaw, C.B., Knox, D.P., and McNeilly, T.N. (2016). A preliminary proteomic characterisation of extracellular vesicles released by the ovine parasitic nematode, *Teladorsagia circumcincta*. Vet. Parasitol. *221*, 84–92.

Vannella, K.M., Ramalingam, T.R., Borthwick, L.A., Barron, L., Hart, K.M., Thompson, R.W., Kindrachuk, K.N., Cheever, A.W., White, S., Budelsky, A.L., et al. (2016). Combinatorial targeting of TSLP, IL-25, and IL-33 in type 2 cytokine-driven inflammation and fibrosis. Sci. Transl. Med. *8*, 337ra65.

Vidal, K., Grosjean, I., evillard, J.P., Gespach, C., and Kaiserlian, D. (1993). Immortalization of mouse intestinal epithelial cells by the SV40-large T gene. Phenotypic and immune characterization of the MODE-K cell line. J. Immunol. Methods *166*, 63–73.

Weng, M., Huntley, D., Huang, I.F., Foye-Jackson, O., Wang, L., Sarkissian, A., Zhou, Q., Walker, W.A., Cherayil, B.J., and Shi, H.N. (2007). Alternatively activated macrophages in intestinal helminth infection: effects on concurrent bacterial colitis. J. Immunol. *179*, 4721–4731.

Zaiss, M.M., Maslowski, K.M., Mosconi, I., Guenat, N., Marsland, B.J., and Harris, N.L. (2013). IL-1β suppresses innate IL-25 and IL-33 production and maintains helminth chronicity. PLoS Pathog. *9*, e1003531.

Zamanian, M., Fraser, L.M., Agbedanu, P.N., Harischandra, H., Moorhead, A.R., Day, T.A., Bartholomay, L.C., and Kimber, M.J. (2015). Release of small RNA-containing exosome-like vesicles from the human filarial parasite *Brugia malayi*. PLoS Negl. Trop. Dis. *9*, e0004069.

Cell Reports, Volume *19*

Supplemental Information

Extracellular Vesicles from a Helminth Parasite Suppress Macrophage Activation and Constitute an Effective Vaccine for Protective Immunity

Gillian Coakley, Jana L. McCaskill, Jessica G. Borger, Fabio Simbari, Elaine Robertson, Marissa Millar, Yvonne Harcus, Henry J. McSorley, Rick M. Maizels, and Amy H. Buck

Figure S1

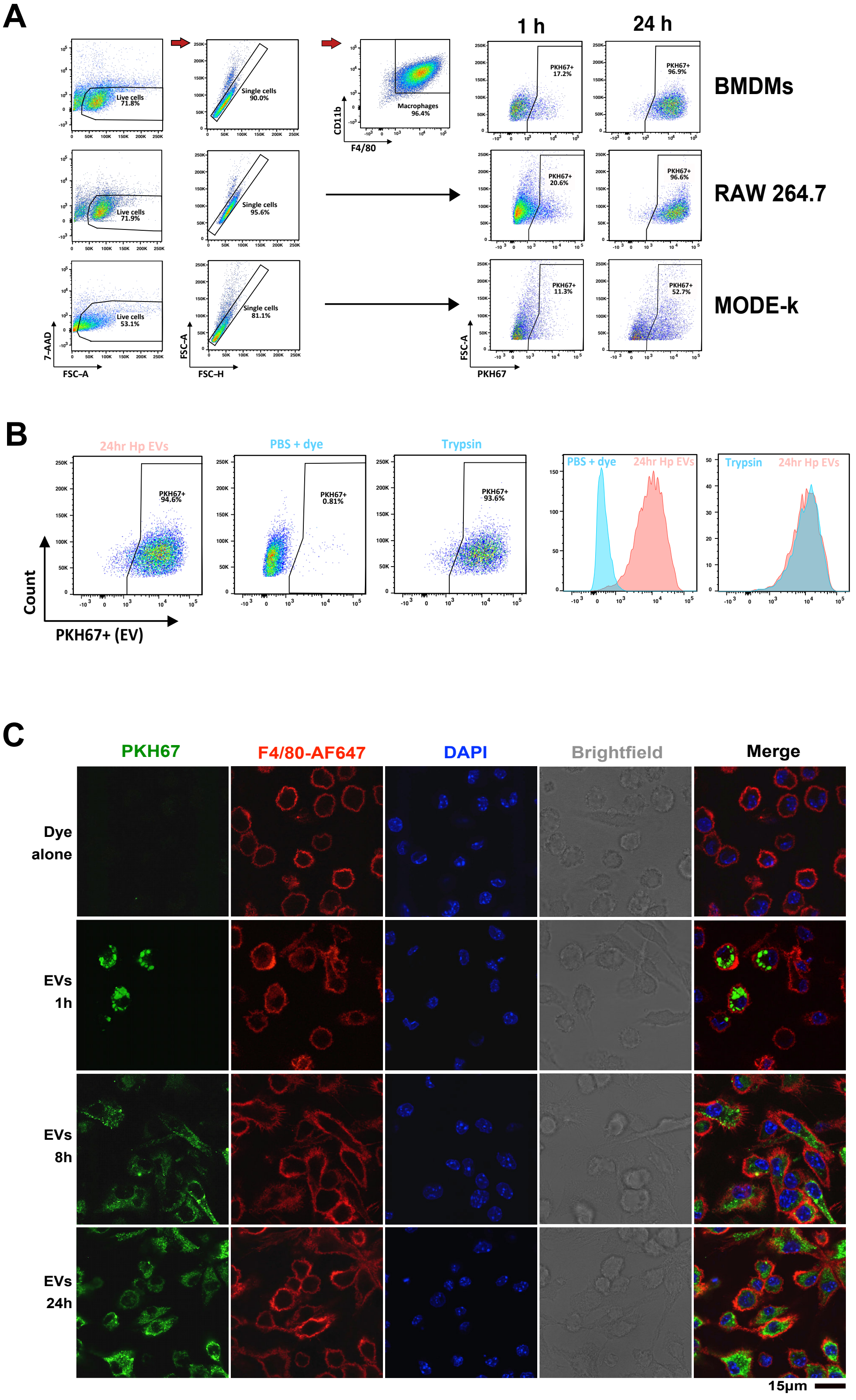

Supplementary Figure 1 - Related to Figure 1
(A) Gating strategy for BMDMs (top), RAW 264.7 (middle) cells and MODE-K cells (bottom), in EV uptake assay, isolating the live, single-cell populations (and F480+CD11b+ for macrophage identity), and subsequent uptake of 2.5 µg PHK67-labelled EVs after a 1 h or 24 h. (B) Gating for control assay comparing 24 h incubation of BMDMs with H. polygyrus PKH67-labelled EVs following trypsin treatment (left) or with the PKH67 dye alone (right). (C) Representative single-channel plots of macrophages incubated with PKH67-labelled EVs or dye alone for 1-24 h, DAPI is used to stain nuclei and F4/80-AlexaFlour 647 labelling shows macrophages. Images were taken using a Leica confocal laser-scanning microscope SP5 using a x63 objective; scale bars indicate 15 µm, with representative images shown from 2 independent experiments.

Figure S2

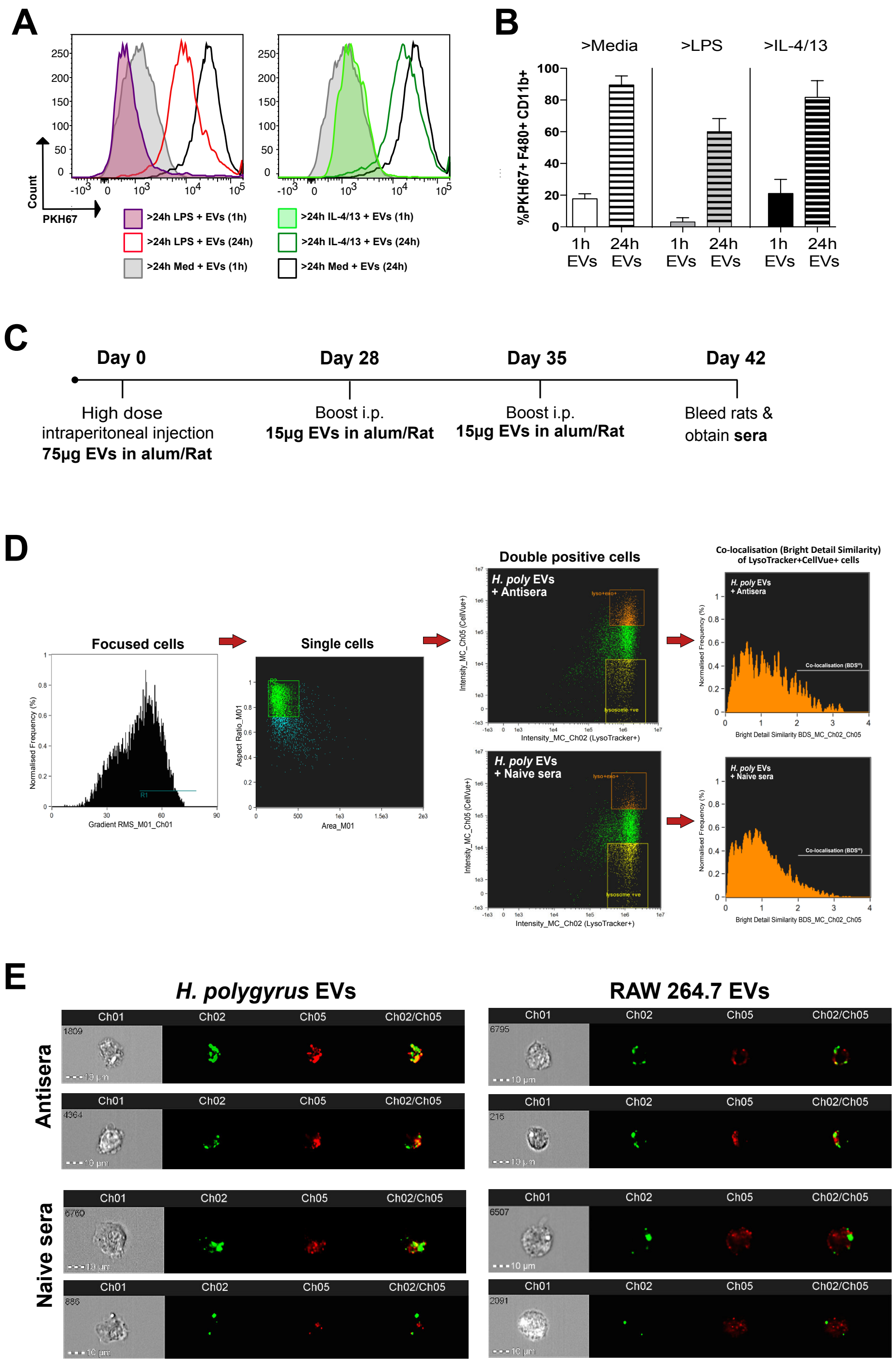

Supplementary Figure 2 - Related to Figure 2

(A) Uptake of 2.5 μg EVs was determined after 1 h or 24 h (as indicated) by shift in fluorescence of PKH67 or, (B) the percentage of PHK67+F4/80+CD11b+ cells, in BMDMs pre-treated with 500 ng/ml LPS, 20 ng/ml IL-4/IL-13 or media alone for 24 h. (C) Immunisation schedule for generation of polyclonal anti-EV sera in rats. (D) IDEAS software analysis of acquired BMDMs gated on "cells in focus", "single cells", and cellular expression of LysoTracker/CellVue, isolating double positive cells to determine their Bright Detail Similarity (histograms in left panel showing the co-localisation between the fluorescent signals of lysosomes and labelled EVs). (E) Further examples of CellVue-labelled *H. polygyrus* or RAW 264/7-derived EVs incubated with 2.5 x 10^5 BMDMs (in the presence of naive sera or antisera), as visualised using imagestream analysis. BMDMs incubated with EVs/sera were stained with LysoTracker Green (n=5 biological replicates with 10,000 live cell images taken per replicate).

Figure S3

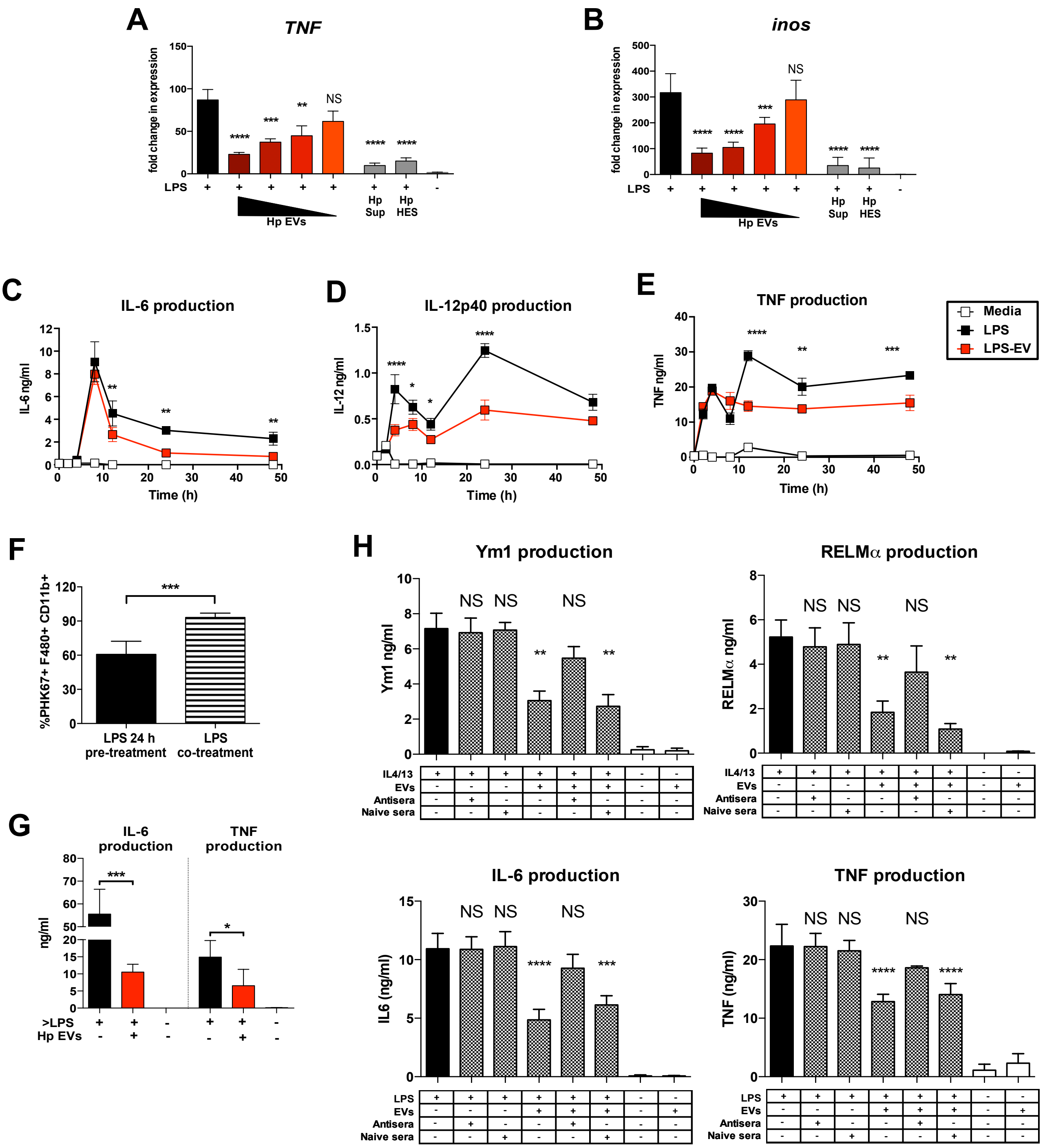

Supplementary Figure 3 - Related to Figure 3

(A, B) Relative expression of Tnf and Inos were measured by qRT-PCR and normalized to GAPDH to determine dose dependent effect of EVs (5, 1, 0.5 and 0.25 µg/ml) compared to 5 µg/ml of Sup or HES on LPS stimulation. (C-E) Supernatant levels of IL-6, IL-12p40 and TNF released over 48 h in BMDMs co-treated with 500ng/ml LPS ± 5 µg/ml EVs as measured by ELISA. (F) Uptake of 2.5 µg EVs was determined after 24 h by assessing the percentage of PKH67+F4/80+CD11b+ cells, in BMDMs pre-treated with 500 ng/ml LPS for 24 h, or at time of EV incubation (as indicated). (G) Levels of IL-6 and TNF assessed by ELISA in BMDMs pre-treated for 24 h (indicated by >) or co-treated with 500 ng/ml LPS ± 5 µg/ml EVs or media. (H) Levels of Ym1, RELMα, IL-6 and TNF assessed by ELISA in BMDMs co-treated with IL4/13 or LPS respectively ± 5 µg/ml EVs or media. (A-H) Data are pooled from 2-3 independent experiments (n=6-8) and presented as mean values ± SD; one-way ANOVA for (A-B) + (F-H); two-way ANOVA for (C-E)). * = $p<0.05$, ** = $p<0.01$, *** = $p<0.001$, **** = $p<0.0001$

Figure S4

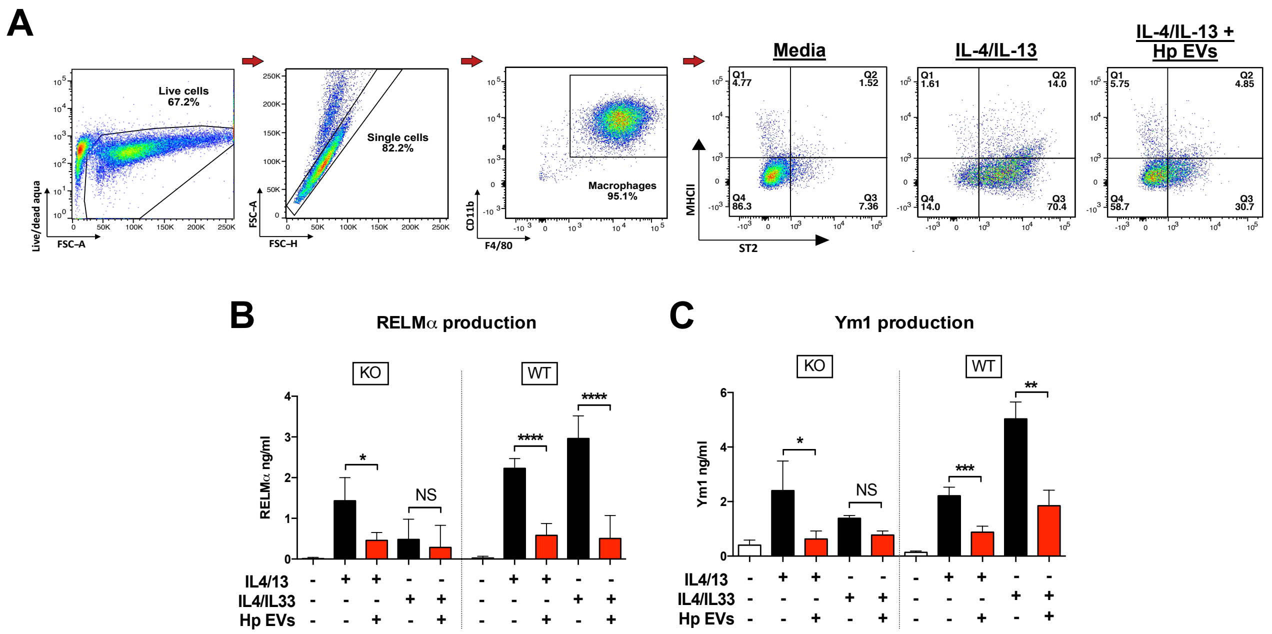

Supplementary Figure 4 - Related to Figure 4

(A) Gating strategy for ST2 expression in IL-4/-13 activated macrophages, denoting live cells (live/dead aqua$^-$), exclusion of doublets, macrophages (F4/80$^+$CD11b$^+$), and activation status (ST2 v MHCII). (B) Levels of RELMα and (C) Ym1 found in supernatant from wild type BALB/c and T1/ST2$^{-/-}$ BMDMs treated with 20ng/ml IL-4/IL-33 and/or IL-4/-13 +/- 5 µg/ml EVs for 24 h as measured by ELISA. Data are pooled from 2 independent experiments (n=6) and presented as mean values ± SD; one-way ANOVA. NS indicates a non-significant result. * = p<0.05, ** = p<0.01, *** = p<0.001, **** = p<0.0001.

Figure S5

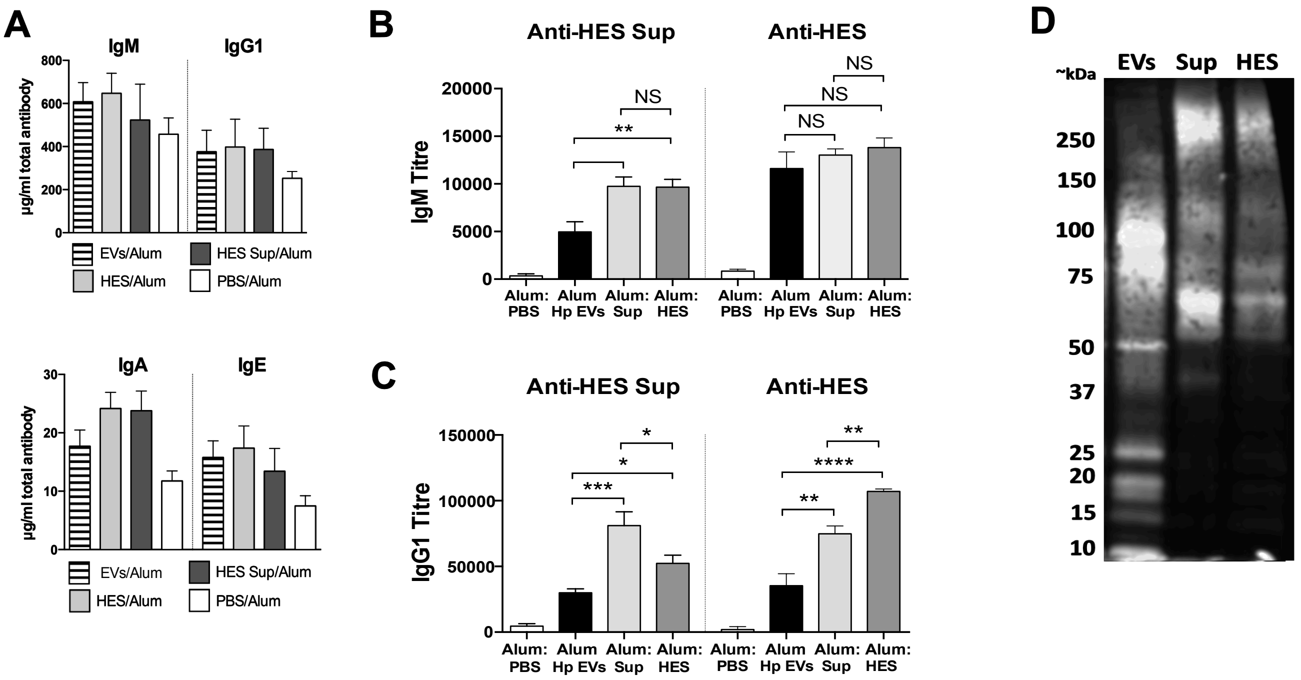

Supplementary Figure 5 - Related to Figure 5

Aged matched female C57BL/6 were vaccinated with EVs, Sup, HES or PBS in alum adjuvant (as described in methods). (A) Total serum IgM, IgG1, IgA and IgE levels in each vaccinated group were determined by ELISA against a standard curve of recombinant mouse IgM, IgG1, IgA or IgE. Data are representative from 1 of 2 experiments (n = 5) and presented as mean values ± SD. (B-C) HES-specific and Sup-specific IgM and IgG1 serum titres from EV, Sup, HES or PBS immunized mice (no challenge infection) were measured by ELISA. Data are pooled from 2 experiments and presented as mean values ± SD (n = 10 mice per group). (D) Representative western blot analysis for reactivity of rat polyclonal sera against 2 µg EVs, HES supernatant or total HES, which had first been resolved by SDS-PAGE.

Figure S6

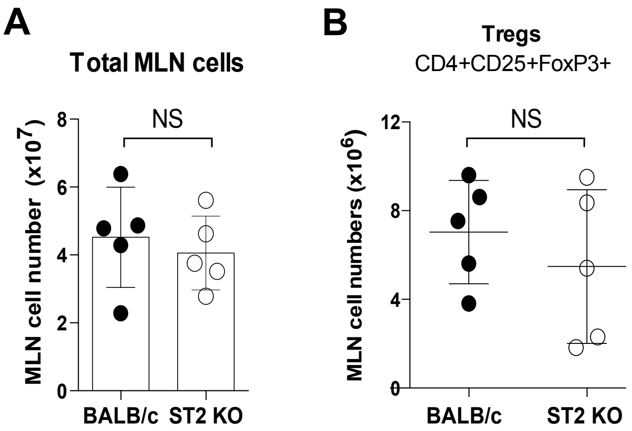

Supplementary Figure 6 - Related to Figure 6

MLNs recovered from *H. polygyrus*-infected BALB/c and T1/ST2$^{-/-}$ mice were isolated at day 28 post-infection. (A) Total numbers of MLN cells recovered or (B) absolute numbers of T regulatory cells (CD4+CD25+FoxP3+) were determined by flow cytometry. Data are representative from 1 of 2 experiments and presented as mean values ± SD (n = 5 mice per group; Student's t test). NS indicates a non-significant result.

Figure S7

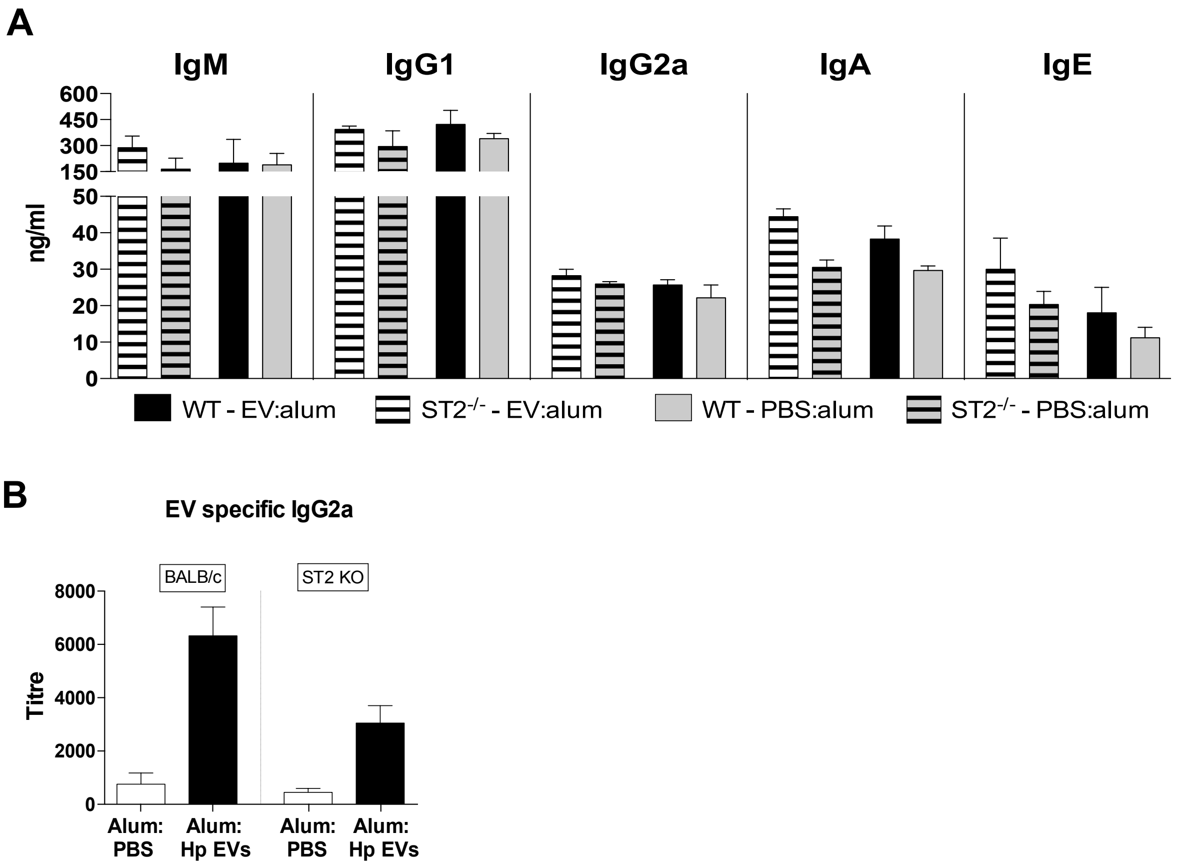

Supplementary Figure 7 - Related to Figure 7

Aged matched female BALB/c and T1/ST2$^{-/-}$ mice were vaccinated with EVs or PBS in alum adjuvant (as described in Figure 5A). (A) Total serum IgM, IgG1, IgG2a, IgA and IgE levels in each vaccinated group were determined by ELISA against a standard curve of recombinant mouse IgM, IgG1, IgG2a, IgA or IgE. (B) EV-specific titres of IgG2a detectable from sera of EV or PBS immunized mice measured by ELISA. Data are representative from 1 of 2 experiments and presented as mean values ± SD (n = 4 mice per group).

Supplemental Experimental Procedures

Enzyme-linked immunosorbent assay

Supernatants from BMDM culture or *ex vivo* lymphocyte re-stimulation experiments were analysed for levels of IL-13 and IFNγ by ELISA using monoclonal capture and biotinylated detection antibody pairs (BD bioscience). Plates were developed with streptavidin-alkaline phosphatase and p-nitrophenyl phosphate substrate (both Sigma), and read at 405nm.

Primer list for Quantitative Real-time PCR

Primer	Target mRNA sequence	5'- 3' sequence
Arg-1 FWD	Arginase	GTC TGT GGG GAA AGC CAA T
Arg-1 REV		GCT TCC AAC TGC CAG ACT GT
GAPDH FWD	Housekeeping gene	CAT GGC CTT CCG TGT TCC TA
GAPDH REV		GCG GCA CGT CAG ATC CA
IL1R1 FWD	IL1 receptor-like 1	AGA CCT GTT ACC TGG GCA AG
IL1R1 REV		CAC CTG TCT TCT GCT ATT CTG G
IL6 FWD	IL6 cytokine	TGC CTT CAT TTA TCC CTT TGA A
IL6 REV		TTA CTA CAT TCA GCC AAA AAG CAC
iNOS FWD	Nitric oxide synthase	CAG CTG GGC TGT ACA AAC CTT
iNOS REV		CAT TGG AAG TGA AGC GTT TCG
RELMα FWD	Resistin-like molecule-α	TAT GAA CAG ATG GGC CTC CT
RELMα REV		GGC AGT TGC AAG TAT CTC CAC
TNF FWD	Tumour necrosis factor-α	GGA AAT AGC TCC CAG AAA AGC AAG
TNF REV		TAG CAA ATC GGC TGA CGG TGT G
Ym1 FWD	Murine chitinase	CAT GAG CAA GAC TTG CGT GAC
Ym1 REV		GGT CCA AAC TTC CAT CCT CCA

Western blot analysis

To test the affinity of EV antisera, 5 µg of whole EVs, HES or HES depleted of EVs (HES Sup) were used. Samples were denatured and separated on NuPAGE 4−12% Bis–Tris gels using NuPAGE MES running buffer (Invitrogen) for 35 min at 200 V, before transfer to a nitrocellulose membrane (Biorad) by running in NuPAGE Western buffer (Invitrogen) for 90min at 120 V. Blots were blocked in 5% Milk-PBS with 0.05% Tween 20 for 2 h at RT, before primary polyclonal antisera probe overnight at 4 °C. Using the Licor-based fluorescence detection system, membranes were incubated with a 1:10,000 dilution of Alexa Fluor-680 conjugated to goat anti-Rat IgG (Thermo Fisher Scientific) in 5% milk-PBSt for 45 min, washed in PBS-tween and acquired in PBS using the Odyssey Scanner (Li-Cor Biosciences).
